# Supplementary material for: Sources of gene expression variation in a globally diverse human cohort
Source: Nature. 2024 Jul 17;632(8023):122–30. doi: 10.1038/s41586-024-07708-2 (PMC11291278; doi:10.1038/s41586-024-07708-2)
Supplement: Supplementary file 1 — This file contains supplementary methods, Supplementary Figs. 1–21 and references. [file 41586_2024_7708_MOESM1_ESM.pdf]

nature portfolio

https://doi.org/10.1038/s41586-024-07708-2

Supplementary information

Sources of gene expression variation in a globally diverse human cohort

In the format provided by the authors and unedited

Supplementary Information

Sources of gene expression variation in a globally diverse human cohort

Taylor *et al.*, 2024

Supplementary Methods: 3
1 Study design 3
2 Ancestry analysis 3
3 RNA sequencing data production 5
3.1 Cell line processing and shipping 5
3.2 RNA extraction and sequencing 5
4 Preliminary gene expression level quantification 6
4.1 Gene-level counts 6
4.2 Lowly-expressed gene filter 6
5 Preliminary alternative splicing quantification 6
5.1 Quantification of intron excision ratios 6
5.2 Filtering lowly-expressed and low-complexity clusters 7
6 Quantifying the contribution of batch effects to expression variation 8
7 Differential gene expression between populations 10
7.1 Data preparation 10
7.2 Factor contrasts 10
8 Expression level variation within and between populations 10
8.1 Normalized expression matrix 10
8.2 Estimation of biological variation 11
9 Splicing variation within and between populations 12
10 *cis*-eQTL mapping 13
10.1 Expression normalization 13
10.2 Calculation of genotype PCs 13
10.3 Calculation of PEER covariates 14
10.4 Discovery of nominal *cis*-eQTLs with FastQTL 16
10.5 Fine-mapping eGene credible sets with SuSiE 17
10.6 Comparison of fine-mapping resolution in subsets of MAGE 17
10.7 Calculation of Allelic Fold Change (aFC) 18
11 *cis*-sQTL mapping 19
11.1 Splicing normalization 19

11.2 Calculation of PEER covariates ... 19
11.3 Discovery of nominal *cis*-sQTLs with FastQTL ... 19
11.4 Fine-mapping sGene credible sets with SuSiE ... 20
12 Analysis of negative selection ... 20
13 Functional annotation and enrichment of fine-mapped *cis*-QTLs ... 21
13.1 Functional annotation of *cis*-eQTLs ... 21
13.2 Functional annotation of *cis*-sQTLs ... 27
14 Colocalization of *cis*-QTLs with complex trait GWAS ... 27
15 Lead e- and sQTL AF differentiation between populations ... 29
16 Replication of credible sets in GTEx ... 29
16.1 Defining replicating vs. non-replicating eQTLs ... 29
16.2 Functional annotation and enrichment of non-replicating eQTLs ... 29
17 Relationship between fixation index and differential gene expression ... 32
18 *cis*-eQTL effect size heterogeneity between populations ... 32
18.1 Modeling interaction effects between genotype and continental group ... 32
18.2 Stratified *cis*-eQTL mapping and effect size estimation ... 33
References: ... 36

Supplementary Methods:

1 Study design

RNA sequencing was performed entirely by GENEWIZ, from Azenta Life Sciences in South Plainfield, New Jersey. We performed RNA sequencing of 779 cell lines. These cell lines represent 731 unique samples, 24 of which were sequenced in triplicate. Sequencing was performed in batches of 15-48 cell lines each (twelve batches of 48 cell lines, four batches of 47 cell lines, and one batch of 15 cell lines). For samples with replicates, replicates were divided between batches such that one replicate of the three was sequenced in one batch, and the other two replicates were sequenced in a separate batch to allow for analysis of inter- and intra-batch variation for each of these samples.

2 Ancestry analysis

Ancestry composition of our study sample was assessed and compared to related studies using ADMIXTURE (version 1.3.0)[58], which uses a likelihood model to estimate allele frequencies in *k* postulated ancestral populations, as well as ancestry proportions for each individual that trace to each of those *k* populations. Genotype data for 1000 Genomes Project[6] (1KGP) samples were obtained from published data based on high coverage (~30×) sequencing by the New York Genome Center (NYGC)[24], subsetting to samples used in our study, by the Geuvadis consortium[4], and or the African Functional Genomics Resource[17] (AFGR). We note that 10 samples from Geuvadis and 2 AFGR samples were not included in the NYGC 1KGP VCF and these samples were excluded from our analysis. Data were downsampled to SNPs in approximate linkage equilibrium (using the `--indep-pairwise 200 20 0.2` flag in PLINK[62]) and restricted to common variants with MAF > 0.05 within the sample. This set of variants was then extracted from genotype data from v9 of the GTEx Project[26] as well as genotype data for samples from the Maasai (MKK) population from AFGR (which are not part of 1KGP), requiring that the SNPs be polymorphic and biallelic (with the same two alleles) in all data sets. One sample from GTEx was not included in the GTEx v9 VCF and this sample was excluded from our analysis. Genotype data from this subset of variants was then merged across the relevant data sets and used as input to ADMIXTURE with default stopping criteria. For the purpose of visualization, *k* was set to 7, which exhibited the minimum 5-fold cross-validation error for the tested range of *k* = [2 .. 10] (**Fig. S1**). ADMIXTURE plots for the MAGE samples for each of *k* = [3 .. 9] are shown in **Fig. S2**. Principal components analysis was performed on the same merged data set using PLINK (version v1.90b6.21)[62] (**Fig. S3**).

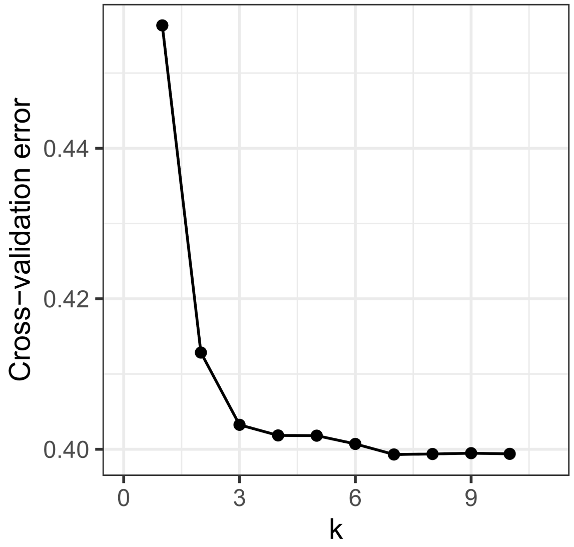

Figure S1. ADMIXTURE cross validation error. Five-fold cross-validation error with varying numbers of specified ancestry components (k) in ADMIXTURE. We selected k=7 for use in **Fig. 1D** as this value minimizes the cross-validation error.

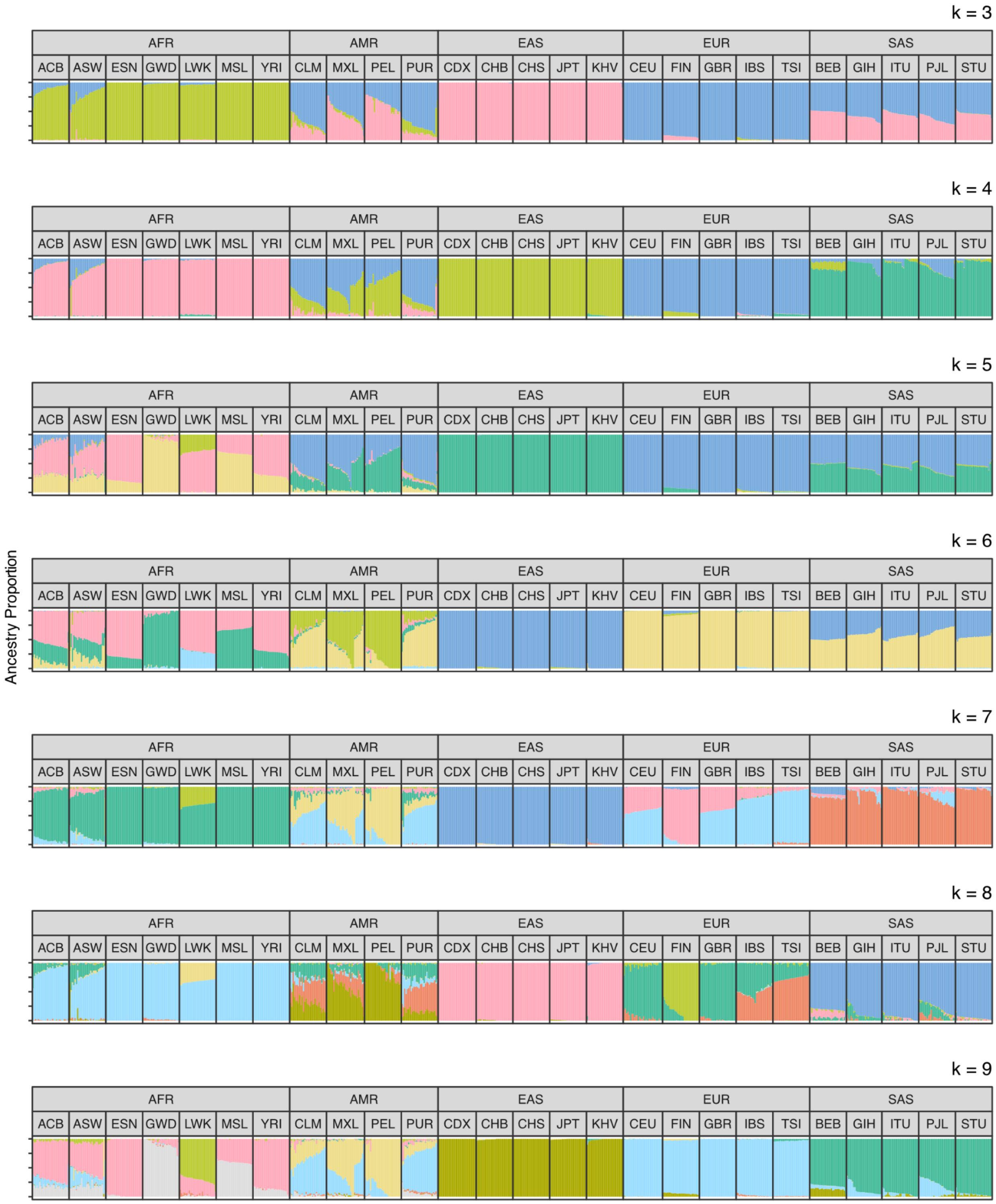

Figure S2. ADMIXTURE results across a range of k. ADMIXTURE results for samples in MAGE displaying proportions of individual genomes (columns) attributed to varying numbers of specified ancestry components (k).

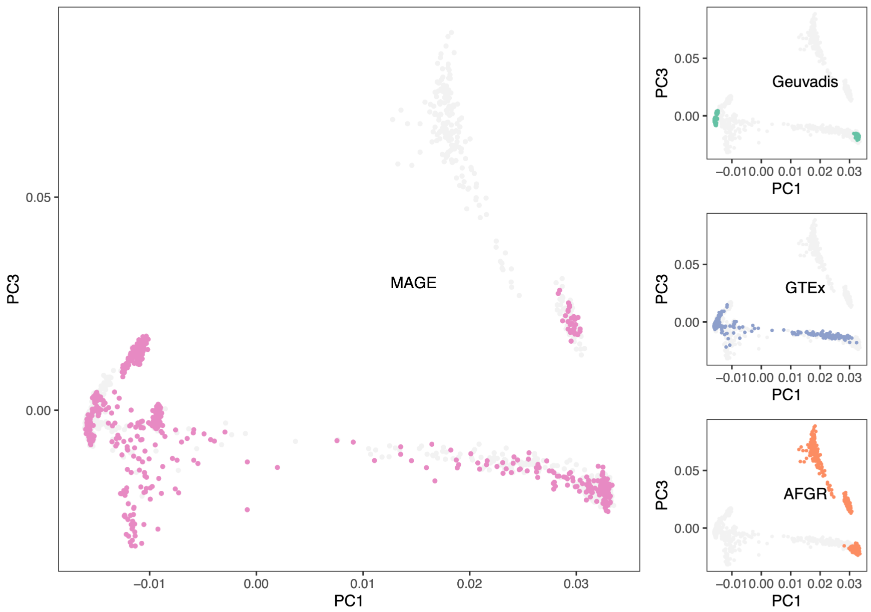

Figure S3. Principal components analysis of genotype data corresponding to various human RNA-sequencing genomic data sets. (A) Genotype principal components 1 and 3 with samples from all studies depicted with gray points and samples from the specified study (i.e., MAGE [pink], Geuvadis [green], GTEx [blue], and AFGR [orange]) depicted with colored points in each respective panel. Note that principal components 1 and 2 are visualized in **Fig. 1B**. The percent of genetic variance explained by principal component 3 is visualized in **Fig. 1C**.

3 RNA sequencing data production

3.1 Cell line processing and shipping

EBV transformed lymphoblastoid cells lines (LCLs) were purchased from the Coriell Institute for Medical Research (NIGMS and NHGRI Repositories) in Camden, New Jersey. All cell lines are free of bacterial, fungal, or mycoplasma contamination. Frozen cell pellets ($\geq$ 5 million cells per cell line) were recovered by Coriell and cultured for 4 days (see Coriell LCL culture FAQ for information about growth media: www.coriell.org/0/sections/support/global/Lymphoblastoid.aspx). After growth, cells were transferred to a growth-limiting shipping media and were shipped directly to GENEWIZ (same-day delivery) for RNA isolation, library prep, and sequencing.

3.2 RNA extraction and sequencing

At Coriell, cells were spun down, then total RNA was extracted from cell pellets using Qiagen RNeasy Plus Universal mini kit following manufacturer's instructions. RNA was quantified using Qubit 2.0 Fluorometer and RNA integrity was checked using the Agilent TapeStation. Sequencing libraries were prepared using the unstranded NEBNext Ultra II RNA Library Prep Kit for Illumina using manufacturer's instructions with the polyA enrichment workflow. Sequencing libraries were validated on the Agilent TapeStation and quantified by using Qubit 2.0 Fluorometer as well as by quantitative PCR. Sequencing was done on the Illumina NovaSeq 6000 instrument with 150 bp paired-end sequencing, with a desired minimum depth of 25M reads per sample. Sequencing libraries were multiplexed in batches of 15-48 samples (the same batches they were shipped in) and loaded onto the flow cell according to manufacturer's instructions.

4 Preliminary gene expression level quantification

4.1 Gene-level counts

To quantify gene expression level in our data set, we use the GENCODE v38 transcript annotations[63] and Salmon (version 1.5.2)[64] for expression quantification. Salmon is a kmer-based method that uses raw RNA-seq data to estimate the number of reads aligning to a defined set of transcripts and their relative abundance. We first generated a Salmon index using `salmon index` with the GENCODE v38 transcript FASTA file as input and with the `--gencode` flag. For each of the 779 cell lines, we quantify transcript-level expression using `salmon quant` with the raw RNA-seq reads as input. We set `--libType=IU` because our sequencing pipeline is expected to produce read-pairs that are inwardly-oriented and unstranded. All other arguments use their default values. This produces, for each library, transcript-level estimates of read counts and TPM. Finally, these transcript-level estimates were summed to gene-level estimates using tximport (version 1.18.0)[65] in R. These gene-level quantifications are used as a starting point in down-stream analyses. Unless otherwise stated, for the 24 samples that were sequenced in triplicate, downstream analyses are limited to the replicate with the most reads for each of these samples.

4.2 Lowly-expressed gene filter

For most analyses of gene expression level differences, it is useful to filter out genes with low expression across samples. Expression quantifications for lowly-expressed genes may be indistinguishable from sequencing noise and can introduce false-positive results across analyses. As such, we limit most analyses to genes with ≥6 counts and ≥0.1 TPM in at least 20% (147/731) of samples. After filtering, we were left with 20,154 expressed genes (19,539 autosomal genes, 615 genes on chrX) used for analyses of gene expression level.

5 Preliminary alternative splicing quantification

5.1 Quantification of intron excision ratios

To quantify alternative splicing in our data set, we followed the splicing quantification pipeline developed by the GTEx consortium and described in their paper[26] and on their GitHub repository (https://github.com/broadinstitute/gtex-pipeline/tree/master/qtl/leafcutter). Briefly, reads were first aligned to the reference with STAR (version 2.7.10a)[66], using WASP correction to mitigate allelic mapping bias. For WASP correction, we used phased variant calls from the NYGC's high-coverage sequencing of the 1KGP[24] (20201028 accession, located on the International Genome Sample Resource[67] ftp server here: https://ftp.1000genomes.ebi.ac.uk/vol1/ftp/data_collections/1000G_2504_high_coverage/working/20201028_3202_phased/). All other options were as described by GTEx.

Previous studies (e.g., [68,69]) have explored different strategies for mitigating reference bias, finding that "ancestry-matched" reference genomes yield only modest benefits. This finding is intuitive given the distribution of common genetic variation, which is largely shared across human populations. Meanwhile, rare variation will be poorly captured in an ancestry-matched reference, as by definition it is unlikely to be shared by another individual, regardless of their membership in any broad ancestry group. Consistent with these conclusions, we observe no systematic difference in the percentage of unmapped reads among samples from different populations, despite the fact that the reference genome used in this study (GRCh38) is primarily derived from a single donor individual (RP-11) of African American ancestry (**Fig. S4**).

Alignments were then supplied to Leafcutter (version 0.2.9)[18] to generate intron excision ratios. Notably, Leafcutter is annotation agnostic; it defines its own splicing "clusters" (groups of related intron excision events) using split reads rather than quantifying splicing using prior exon or transcript annotations. In a data set such as ours, where many individuals are

from historically understudied populations, this eliminates bias from annotations generated from sample sets with limited diversity and may allow us to elucidate novel intron excision events.

Intron usage was estimated for each library using `regtools junctions extract` (regtools version 0.5.2) using a minimum anchor length of 8 bp (`-a 8`), strand specificity set to unstranded (`-s 0`) based on our library prep, minimum intron size set to 50 bp (`-m 50`), and the maximum intron size set to 500kb (`-M 500000`). Junction files were then used to cluster introns across all samples using the Leafcutter `leafcutter_cluster_regtools.py` companion script, where 50 split reads were required to support each cluster (`-m 50`) and the maximum intron size was set to 500kb (`-l 500000`).

While STAR and Leafcutter were run using all 779 sequencing libraries, unless otherwise stated, for the 24 samples that were sequenced in triplicate, downstream analyses used results from the replicate with the most reads for each of these samples.

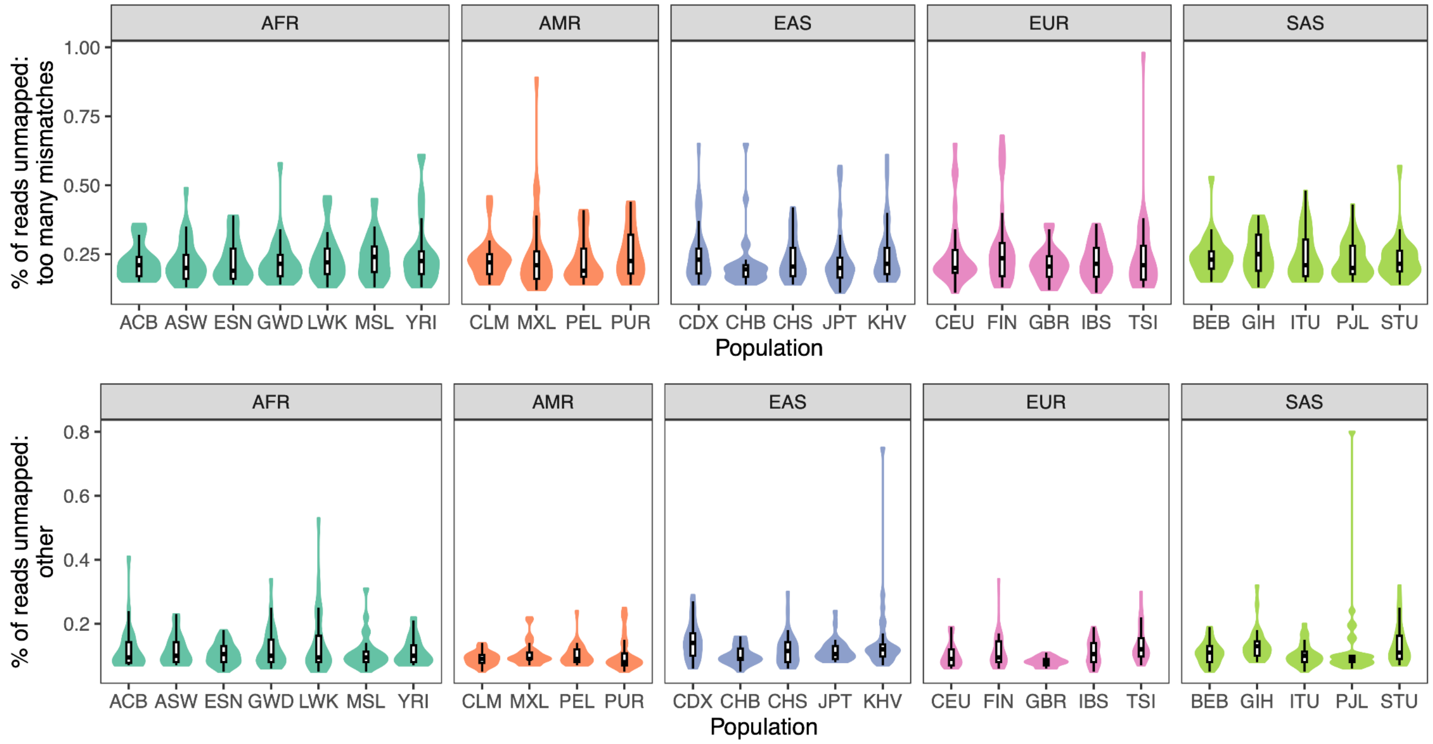

Figure S4. Percentage of unmapped reads per sample, stratified by population. Unmapped reads are separated into reads that were unmapped due to too many mismatches (top row) or unmapped simply because no sufficiently identical reference sequence was identified (bottom row).

5.2 Filtering lowly-expressed and low-complexity clusters

Introns with low counts and clusters with low complexity can lead to statistical issues when discovering sQTLs and quantifying splicing variance. To avoid these issues, we applied a filtering procedure to the intron excision ratios produced by Leafcutter, largely based on the leafcutter filtering applied by GTEx and described in their paper[26] and on their GitHub repository (https://github.com/broadinstitute/gtex-pipeline/blob/master/qtl/leafcutter/src/cluster_prepare_fastqtl.py).

After running Leafcutter, we had intron excision ratios for 245,487 introns (51,466 splicing clusters) on the autosomes and chrX. We first filtered out introns with low complexity across samples, defined as introns without any read counts in >90% of samples, or with fewer than *max(10, 0.1n)* unique values, where $n = 731$ is the sample size. After this step, 154,816 introns (33,712 clusters) remained. Through use of the Leafcutter `prepare_phenotype_table.py` companion script (described in more detail in **section 11.1** below) 8,430 additional introns were dropped with SD < 0.005 across

samples or whose cluster had 0 counts in > 40% of samples. After this step, 146,386 introns (33,447 clusters) remained. Finally, we dropped 580 clusters with only one intron.

After filtering, 145,806 introns (32,867 splicing clusters) were retained and used for down-stream analyses of splicing variation.

6 Quantifying the contribution of batch effects to expression variation

Batch effects–along with other sources of technical variation–are a known confounder in RNA-seq data. As such, it is critical to ensure that the effect of batch on gene expression level does not mask actual biological variation. To assess the contribution of batch effects to expression level variation, we sequenced 24 samples in triplicate, as described in section **1** above. Using the filtered gene-level counts described in section **4.2**, we calculated the Spearman rank correlation between each pair of the 72 replicate sequencing libraries. Critically, we observe that pairs of libraries from the same individual have higher correlations than pairs of libraries from the same batch (**Fig. S5A**).

Additionally, for each of the 19,539 autosomal expressed genes and across the 72 replicate sequencing runs (24 samples sequenced in triplicate), we calculated the proportion of expression level variation explained by sample versus batch. Using a VST normalized expression matrix (described in section **7.1** below) subset to samples sequenced in triplicate, we performed a type II ANOVA using the `Anova` function from the *car* package (version 3.1-2) in R with the following regression formula: *expression ~ batch + sample*. To test whether expression variance between samples was greater than variance measured between sequencing batches, we performed a Wilcoxon signed-rank test using the proportion of variance explained from the ANOVA above. We observed that, on average, the proportion of gene expression variance explained by sample was greater than by sequencing batch (one-tailed Wilcoxon signed-rank test: $p < 1 \times 10^{-10}$), concordant with the results from the Spearman's rank correlation test (**Fig. S5B**).

We performed a complementary set of analyses to quantify the contribution of batch effects to splicing variation. Using the filtered intron excision ratios described in section **5.2**, we calculated the Spearman rank correlation between each pair of the 72 replicate sequencing libraries. As with the analysis of expression level, we observe that pairs of libraries from the same individual have higher correlations than pairs of libraries from the same batch (**Fig. S5C**). Additionally, for each of the 31,837 autosomal splicing clusters that passed filtering, we calculated the proportion of splicing variation explained by sample versus batch. Using the intron excision ratios from Leafcutter, we performed a type II ANOVA using the `manta` function from the *manta* package (version 1.0.0) in R to fit a model that regresses intron excision ratios onto batch and sample. Described in more detail in section **9**, MANTA[70] is a tool for evaluation of multivariate linear models (such as intron excision ratios) that uses the Hellinger distance between splicing ratios to estimate the variability in splicing across individuals. As before, we performed a Wilcoxon signed-rank test using the proportion of variance from MANTA. We observed that, on average, the proportion of splicing variance explained by sample was greater than by sequencing batch (one-tailed Wilcoxon signed-rank test: $p < 1 \times 10^{-10}$) - concordant with the results from the Spearman's rank correlation test (**Fig. S5D**).

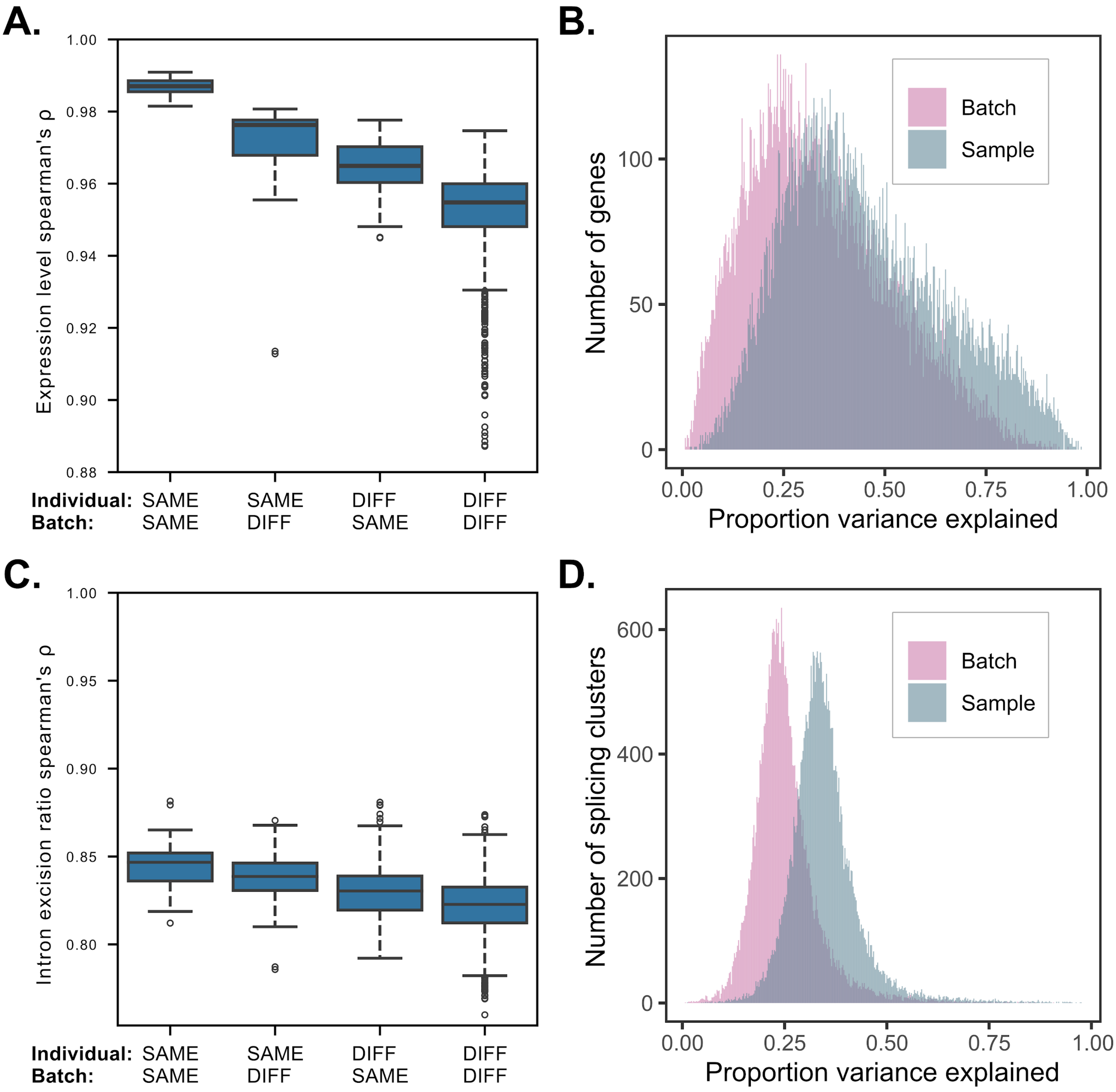

Figure S5. Batch effects in MAGE RNA-seq data. (A) Spearman rank correlation in expression level (TMM) across all expression-filtered genes between each pair of technical replicates in our data set (24 unique samples, 72 total replicates). Pairs of replicates are stratified by 1) whether they were sequenced in the same sequencing batch and 2) whether they were derived from the sample 1KGP sample. Higher correlations are observed for pairs of replicates from the same sample than for pairs of replicates from the same sequencing batch. **(B)** For replicate sequencing libraries and across autosomal expression-filtered genes, per gene estimates of the proportion of variance in gene expression level explained by sequencing batch (pink) or sample (blue). On average, sample explained a higher proportion of variance in expression level than batch one-tailed (Wilcoxon signed-rank test: $p < 1 \times 10^{-10}$). **(C)** Same as panel A, but showing Spearman rank correlation in intron excision ratio across all splicing-filtered introns. Again, higher correlations are observed for pairs of replicates from the same sample than for pairs of replicates from the same sequencing batch. **(D)** Same as panel B, but showing the proportion of variance explained by batch or sample across autosomal splicing-filtered splicing clusters. On average, sample explained a higher proportion of variance in splicing than batch (one-tailed Wilcoxon signed-rank test: $p < 1 \times 10^{-10}$).

7 Differential gene expression between populations

7.1 Data preparation

Differential gene expression (DGE) analysis was performed using *DESeq2* (version 1.36.0[71]) in R. Using the salmon-generated pseudocount expression data per sequence library (detailed in section **4.1**), transcript-level abundances were first converted into gene-level abundances using the *tximport* (version 1.24.0)[65] in R under default parameters. Gene-level expression estimates were imported into the DESeq2 ecosystem with the design formula specified as ~ *population* + *batch* + *sex,* where *batch* and *sex* were included as categorical covariates to control for technical variation between sequencing batches (see section **6**) and sex-dependent effects. For this analysis, technical replicates for each of the 24 samples that were sequenced in triplicate were collapsed into single samples using the `collapseReplicates` function included in DESeq2.

7.2 Factor contrasts

Differential expression contrasts were constructed one of two ways: 1) each population's expression was contrasted against the average expression across all other populations within their parent continental group (e.g., JPT samples vs. all other samples within the East Asian continental group), or 2) each continental group's expression was contrasted against the average expression across all other continental groups (e.g., AFR samples vs. all other samples).

Using the design formula specified in **section 7.1**, Wald test contrast coefficient matrices were extracted for each population by computing the mean coefficients for each dummy variable using all samples within the focal population label (e.g., JPT). For each continental group and background population (e.g., all non-focal subpopulations per continental group), coefficient matrices were additively combined and normalized to the number of populations contained within their respective continental group (yielding a contrast coefficient matrix where the intercept weight = 1). Multiple testing correction, independent filtering, and outlier detection for each contrast were all performed using default functions included in the *DESeq2* package.

8 Expression level variation within and between populations

8.1 Normalized expression matrix

The relationship between populations and expression variance was measured using the blind variance stabilizing transformation (VST) function included in *DESeq2* (version 1.36.0)[71] on the gene-level count matrix produced after collapsing technical replicates (see section **7.1**). VST produces an expression matrix which directly captures the effects of library- and experiment-wide normalization factors, estimated gene-wise dispersion, and reduces the dependence of expression variance on the mean expression per-gene (see **Fig. S6**). This transformed count matrix was reduced to only represent the filtered subset of genes described in section **4.2**.

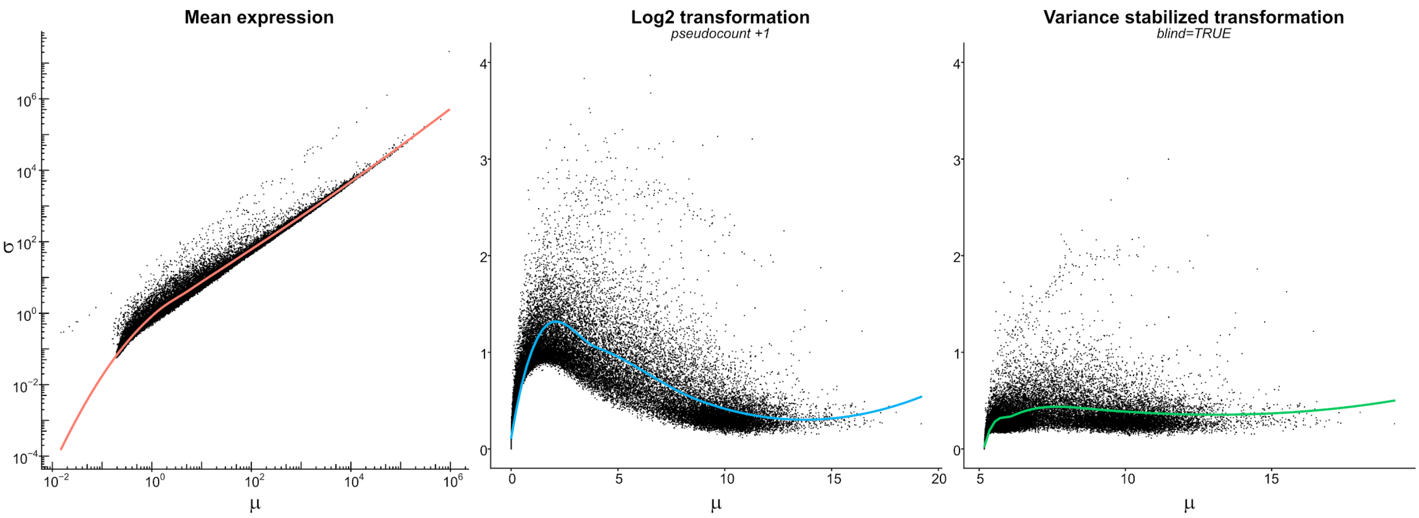

Figure S6. Count-data transformations for examining global trends in gene expression. Experiment-wide expression mean, μ, and standard deviation, σ, computed across all samples following three methods of expression normalization: mean expression computed by DESeq2 after correcting for dispersion and normalization factors (left), Log_2-transformed counts (middle), and variance-stabilized transformation (VST; right).

8.2 Estimation of biological variation

We applied a two-stage ANOVA strategy to quantify gene expression variance at global, continental group, and population scales. First, an ANOVA was performed for each gene using the `anova` and `lm` functions in the *stats* package (version 4.3.0) in R with formula **[1]** below, where *batch* and *sex* were included as categorical covariates to remove technical variance from the response variable. Here u is the residuals of the regression and represents the VST normalized expression values corrected for the effects of batch and sex. Because continental group and population together form a multicollinear system, two independent ANOVAs were then performed to estimate the proportion of gene expression variance due to continental group (formula **[2]**) and population label (formula **[3]**), where the batch- and sex-corrected expression values, u, were used as the response variable. The proportion of variance explained (PVE) was estimated as the regression sum of squares divided by the total sum of squares for each regression. In this manner, the PVE by continental group or by population represent the proportion of variance in the batch- and sex-corrected expression values explained by the label.

$$\textbf{(1)}\quad VST\ Expression \sim sex + batch\ + u$$

$$\textbf{(2)}\quad u \sim continental\ group\ +\ v_1$$

$$\textbf{(3)}\quad u \sim population\ +\ v_2$$

To test whether the variance explained by continental group/population was greater than that expected by chance, we performed a permutation test where continental group and population labels were shuffled (with replacement) and the ANOVA procedure described above was recalculated for each gene (n = 1000 permutation replicates). A permutation test p-value was computed as the proportion of permutations where the mean proportion of variance explained by continental group/population was more extreme (i.e., greater) than those respectively measured in our empirical data set. For both continental group and population, none of the permutations had a mean PVE greater than calculated with the empirical data set.

To quantify variance in gene expression within each continental group, we first applied the same regression strategy to remove variance due to sex and batch from the VST gene expression array using formula **[1]**. For each continental group, and for each gene, residuals were partitioned to include only samples within the focal group, and sample variance was calculated using the `var` function in R.

Using the gene-wise variance estimates per continental group, we tested whether gene expression variance differs significantly across continental populations (**Fig. S7A**). To achieve this objective, we fit a linear mixed model (`lme`, *lme4* package version 1.1-34 in R) to the expression data, where the response variable ($\log_{10}$-transformed variance) was regressed against a continental group fixed-effect and gene included as a random-effect. The performance of this model was compared with a reduced model (without the continental group fixed effect) using the `anova` function in the *stats* package. This statistical procedure was also applied to test whether splicing differs significantly across continental groups (**Fig. S7B**).

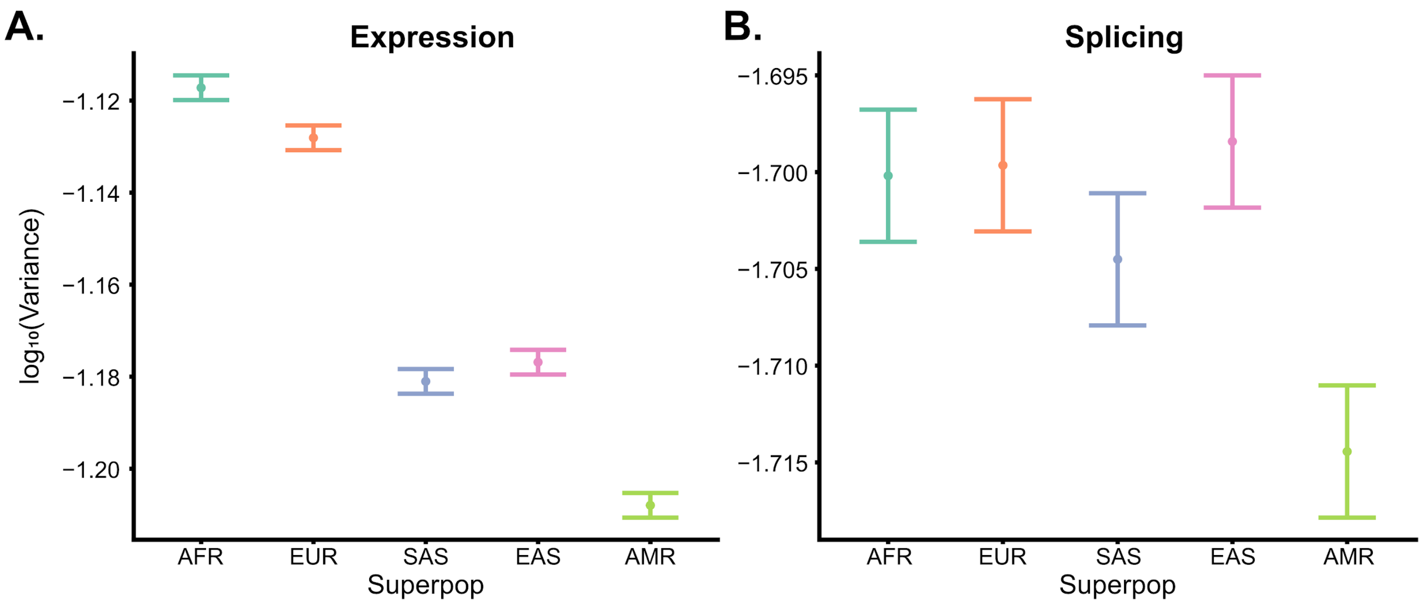

Figure S7. Global trends in gene expression and splicing variance. (A) Variance in gene expression variance decreases with Eastward expansion from Africa. **(B)** In contrast, splicing variance shows little change with Eastward expansion, but decreases significantly in the admixed American continental group relative to the African continental group (two-tailed t-test: p-value = 3.17e-3, n=32,867 splicing clusters). Each point represents the mean variance computed across all genes/splicing clusters within the focal population label, and whiskers represent ± 1 std. Err.

9 Splicing variation within and between populations

The approach to quantify splicing variation between and within continental groups and populations largely mirrors the approach used for gene expression level (detailed in section **8.2**).

As with gene expression level, we applied a two-stage ANOVA strategy to quantify the proportion of splicing variance explained by continental group or population. Because splicing proportions are inherently multivariate, a standard ANOVA is not appropriate. Instead, we used MANTA[70], a tool for evaluation of multivariate linear models including proportion data such as intron excision ratios. MANTA uses the Hellinger distance between splicing ratios to estimate the variability in splicing across individuals.

For each splicing cluster that passed filtering (see section **5.2**), we applied the following procedures. First, we used the `manta` function from the *manta* package (version 1.0.0) in R to regress filtered intron excision ratios onto sample-batch and sex to remove technical variation from the response variable. We set `transform="sqrt"` to use the Hellinger distance between splicing ratios, and `fit=TRUE` to return the regression residuals. Using the residuals from this first step, we then ran two independent ANOVAs to estimate the proportion of splicing variance attributable to continental group and population label. As before, we used the `manta` function to regress the residuals from the first step onto either continental group or population. We did not use the square root transform for these two models, because the residuals from the first step should reflect the initial square root transform. The proportion of variance explained by either

continental group or population was estimated as the regression sum of squares divided by the total sum of squares (after regressing out batch and sex) for each model.

As with gene expression level, we tested whether the variance explained by continental group/population was greater than that expected by chance using a permutation test. Continental group and population labels were shuffled and the above procedure was repeated. 1000 total permutations were performed. We computed a permutation test p-value for both continental group and population as the proportion of permutations where the mean PVE (across splicing clusters) was greater than that calculated from the empirical data set. For both continental group and population, none of the permutations had a mean PVE greater than calculated with the empirical data set.

To quantify variance in splicing within each continental group, we first applied the same regression strategy described above to remove variance from batch and sex from intron excision ratios. For each continental group, and for each splicing cluster, residuals from this regression were partitioned to include only samples within the focal continental group, and sample variance was calculated as:

$$\frac{1}{N(N-1)}\sum_{j}^{N-1}\sum_{k=j+1}^{N} d^2(j,k)$$

where N is the total number of samples within the focal continental group, and $d^2(j,k)$ is the squared Euclidean distance between the residual intron excision ratios (after removing the effects of batch and sex) of the focal splicing cluster for individuals j and k in the focal continental group.

10 *cis*-eQTL mapping

10.1 Expression normalization

Preliminary gene expression counts (described in section **4.1**) were prepared for eQTL mapping using the following procedure: 1) gene-level counts were normalized between samples using TMM[72] as implemented in the *EdgeR* package (version 3.32.1)[73] in R; 2) lowly expressed genes were filtered out (as described in section **4.3**); 3) for each gene that passed filtering, TMM values were inverse normal transformed.

10.2 Calculation of genotype PCs

To control for the effects of global ancestry on gene expression, we first calculated the top 20 genotype principal components (PCs) from the samples included in MAGE. Genotype PCs were computed from the NYGC high-coverage autosomal variant calls (see section **2**) across the 731 samples using PLINK[62] with the `--pca` option and restricting to variants with in-sample MAF > 0.01 using `--maf 0.01`. We observed that the variance explained by consecutive PCs decreased considerably following the first five genotype PCs (**Fig. S8A**). Additionally, the top four genotype PCs correlated strongly with continental group label (**Fig. S8B**), and the top five genotype PCs correlated strongly with population label (**Fig. S8C**). Interestingly, we do observe some weaker correlations with population label; for example, PC10 appears to be correlated with population label, but explains only 0.26% of genotype variance.

Based on these results, the top five genotype PCs were included as covariates in QTL mapping to control for confounding by global ancestry.

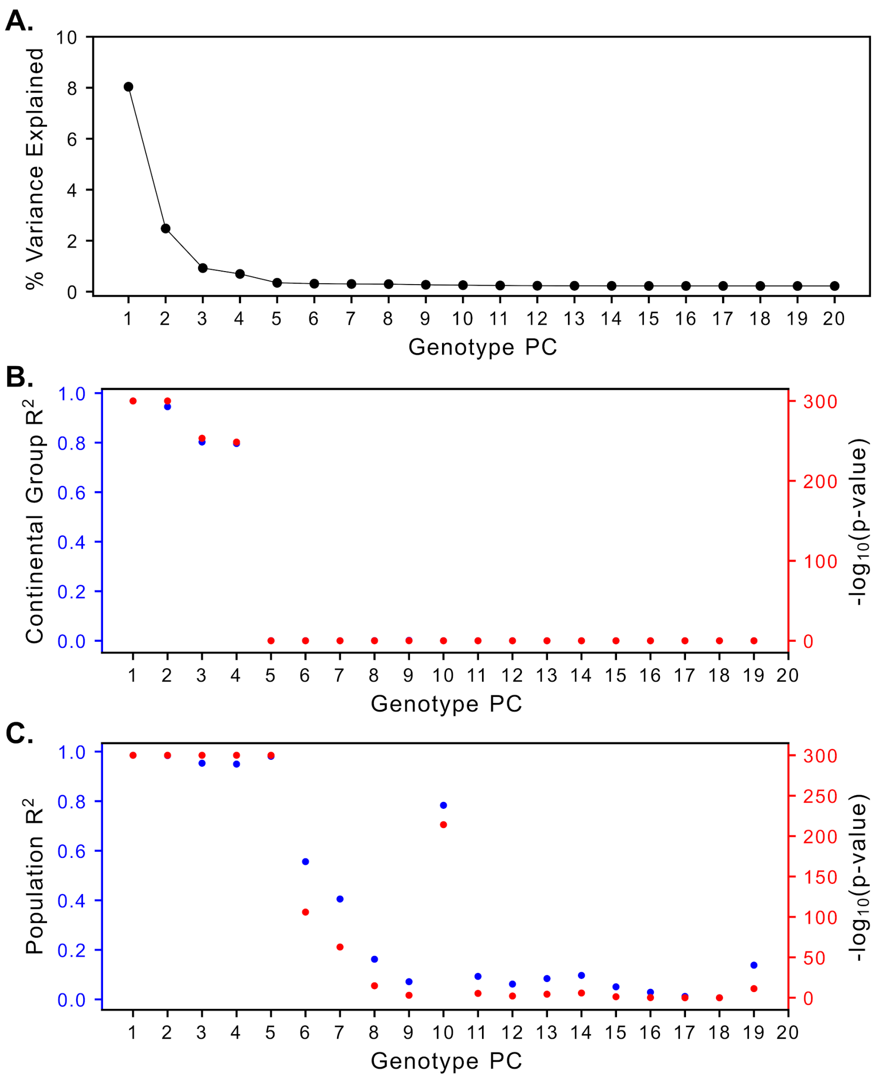

Figure S8. Selection of genotype PCs for QTL mapping. (A) Percent of genotype variance explained by each of the top 20 genotype PCs for the samples in MAGE. Variance explained drops off after 5 PCs. **(B)** Correlation between continental group and each of the top 20 genotype PCs. The first 4 PCs are significantly correlated with sample continental group label. **(C)** Correlation between population label and each of the top 20 genotype PCs. The first 5 PCs are significantly correlated with population label. Interestingly, PC10 also appears to be correlated with population label, but explains only 0.26% of genotype variance. Two-tailed p-values in (B) and (C) are obtained by regressing PCs onto population or continental group.

10.3 Calculation of PEER covariates

Batch effects and other technical sources of variation are known to affect RNA-seq studies and quantification of gene expression and can reduce the power of eQTL mapping if not properly controlled. Because these factors are not necessarily directly measured, we used Probabilistic Estimation of Expression Residuals (PEER)[74] to identify hidden factors driving expression variation in our data set. We chose the number of PEER factors to use as covariates in eQTL mapping based on the optimizations performed previously by GTEx[26,74] Briefly, for each of four sample size bins, GTEx assessed the number of PEER factors that maximized the number of significant e/sGenes discovered. Based on these optimizations, GTEx used 60 PEER factors for eQTL mapping with sample sizes ≥350. To ensure that this was an appropriate selection, we investigated how the number of eGenes varies according to the number of PEER factors within MAGE. We used `peertool` (v1.0) to calculate either 0, 1, 2, 5, 10, 15, 20, 25, 30, 40, 50, 60, 70, 80, or 100 PEER factors from the normalized TMM values (see section **10.1**), limiting the algorithm to 100 iterations using `--n_iter 100`. For each number of PEER factors, we used the FastQTL adaptive permutation mode to discover significant eGenes (see section **10.4** below for details), restricting the analysis to Chromosome 1 for computational efficiency (**Fig. S9**).

We observed a plateau starting at roughly 50 PEER factors whereafter adding additional PEER factors does not substantially increase the number of eGenes discovered. We emphasize that there is no “ground truth” for optimization, and choosing the number of PEER factors to maximize eGene discovery within our data may risk overfitting. Because it falls within the plateau of eGene discovery, and for consistency with GTEx, we chose to use 60 PEER factors as covariates for eQTL mapping.

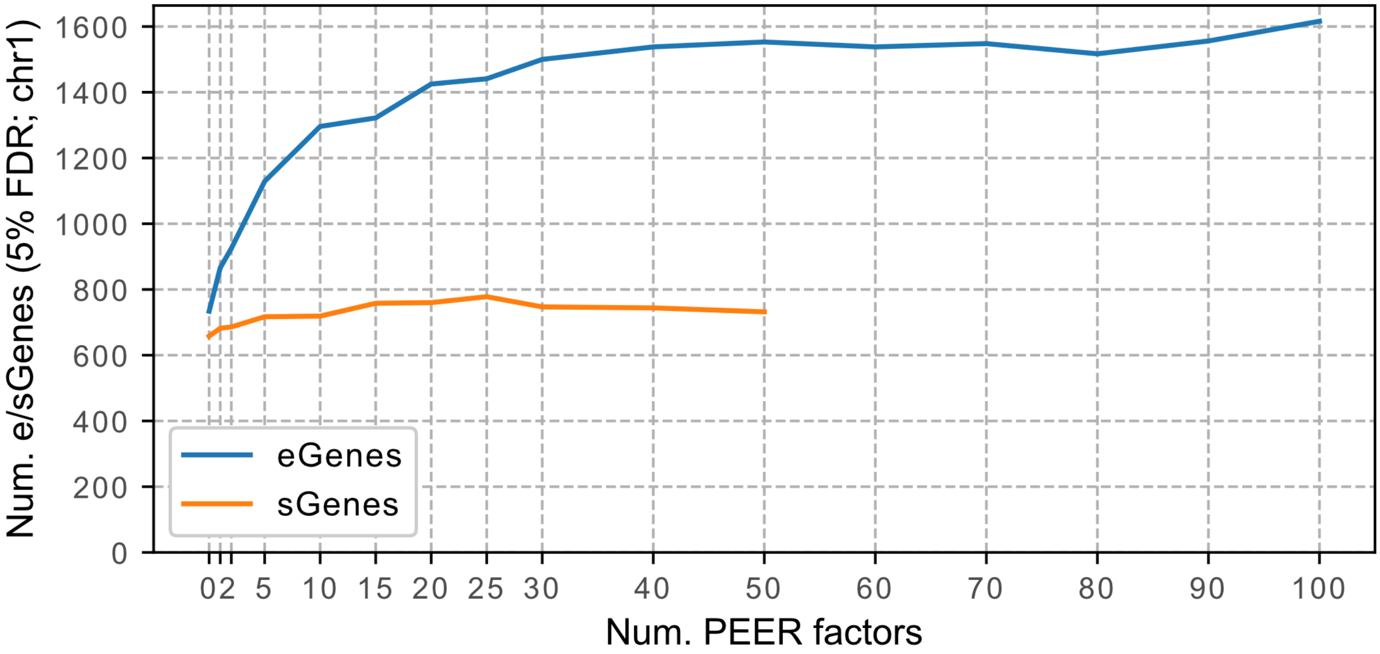

Figure S9. Selection of PEER factors for QTL mapping. Number of eGenes and sGenes on Chromosome 1 discovered with FastQTL using different numbers of PEER factors as covariates. e/sGenes were discovered at a 5% FDR. For eQTL mapping, PEER factors were computed from normalized TMM values from the autosomes and chrX (the same values used as input for eQTL mapping). For sQTL mapping, PEER factors were computed from normalized intron excision ratios from the autosomes and chrX (the same values used as input for sQTL mapping).

We next investigated whether these PEER factors correlated with known confounders in RNA-seq experiments. Specifically, for each PEER factor, we tested its correlation with each of eight confounders: sequencing batch, the study in which the cell lines were first generated (i.e. the International HapMap Project[75], HapMap 3[75,76], or the 1000 Genomes Project[6]), continental group, population, sex, RNA integrity number (RIN), the total number of reads, and the total amount of RNA in the library (**Fig. S10A,B**).

Across PEER factors, we observed the strongest (**Fig. 10A**) and most significant (**Fig. S10B**) correlations with sequencing batch. Batch effects are known confounders in RNA-seq studies, and this observation is not unexpected. For a subset of PEER factors, we also observe weaker, yet significant correlations with sample continental group and population, suggesting that these factors may be picking up some biological variation. We therefore elected not to include these factors as covariates for analyses of differential expression between continental groups/populations (section **8.2**; **Fig. 2A,B**). For the most part however, these factors appear to reflect technical variation between batches.

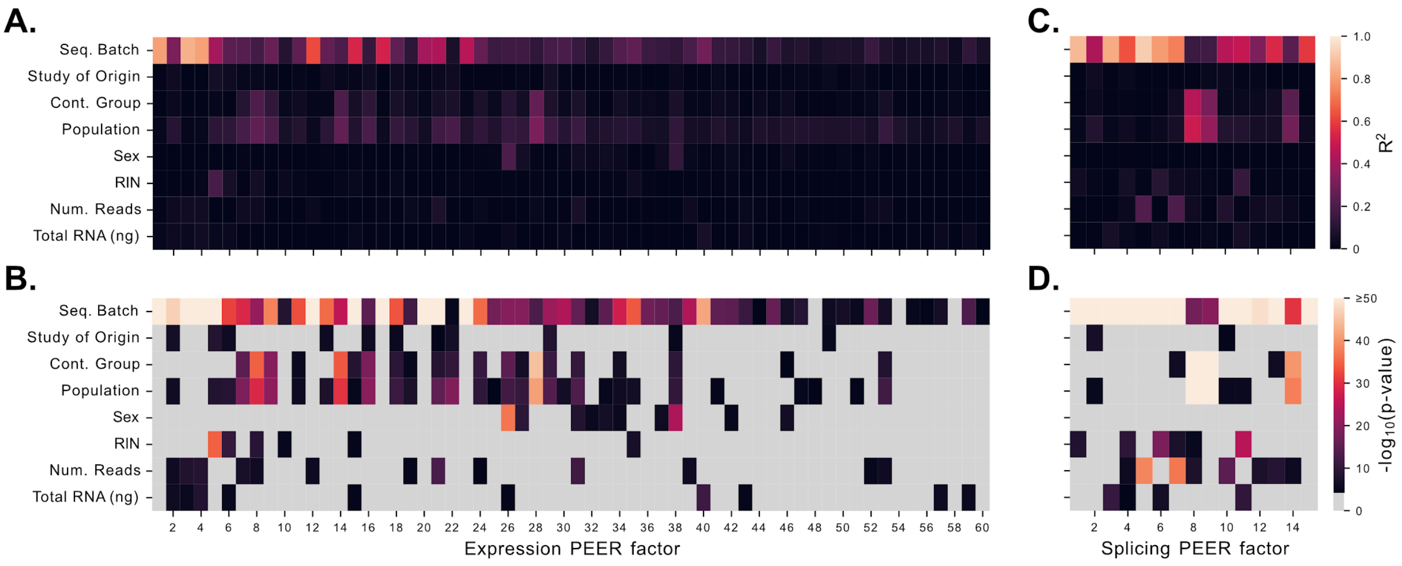

Figure S10. Correlation between PEER factors and known confounders. For each of the 60 expression-derived PEER factors and 15 splicing-derived PEER factors, we tested their correlation with each of eight possible confounders. **(A)** Correlation between the 60 expression-derived PEER factors and the tested confounders. **(B)** The significance of each of the correlations shown in A. Non-significant correlations (based on a Bonferroni threshold of 8.33×10^{-5}) are shown in grey. **(C)** As in A, but for the 15 splicing-derived PEER factors. **(D)** As in B, but for the 15 splicing-derived PEER factors. In (B) and (D), for each covariate, two-tailed p-values in (B) and (D) are obtained by regressing PEER factors onto the covariate.

10.4 Discovery of nominal *cis*-eQTLs with FastQTL

We discovered eQTLs using FastQTL (version v2.184_gtex)[77] as implemented by GTEx (https://github.com/francois-a/fastqtl). For each of the 19,539 autosomal genes that passed filtering thresholds (see section **4.2**), we regressed inverse normal transformed TMM values onto variant genotypes for all variants within 1 Mbp up- and down-stream of the gene's transcription start site (TSS), and with MAF > 0.01. The top 5 genotype PCs (section **10.2**), 60 PEER factors (section **10.3**), and sex were included as covariates.

We also performed *cis*-eQTL mapping for each of the 615 genes on the X chromosome that passed filtering thresholds (see section **4.2**). We note that for the 1KGP variant calls from the NYGC, the pseudoautosomal regions (PARs) on the X chromosome represent both the X chromosome PARs and Y chromosome PARs and as such, all individuals have diploid genotypes in these regions. In the X chromosome non-PARs, XX individuals have diploid genotypes, while XY individuals have haploid genotypes. To enable joint eQTL mapping with XX and XY samples in the non-PARs, we artificially transform XY haploid genotypes to homozygous diploid genotypes in the input VCF file.

Altogether, this approach to handling genotypes on the X chromosome makes two important assumptions about the architecture of gene expression on the X chromosome: 1) genes in the PARs "escape" X-inactivation (i.e. both the X and Y homologs of each gene are expected to be expressed) and 2) genes in the non-PARs are randomly inactivated on one X chromosome homolog in XX samples and are not inactivated in XY samples. While the first assumption is expected to be valid for most genes, some non-PAR genes are known to escape X-inactivation[78] and so caution is advised when interpreting eQTL results for genes in the non-PARs.

We first ran FastQTL in the adaptive permutation mode using `--permute 1000 10000`, to discover significant *cis*-eGenes (genes with at least one cis-eQTL). FastQTL estimates gene-level empirical p-values, based on the theoretical distribution of permutation p-values. The GTEx implementation of FastQTL uses the estimated empirical p-values to calculate gene-level q-values and from these q-values, we discover eGenes at a 5% false discovery rate (FDR) threshold. FastQTL also calculates a nominal p-value threshold for significance for each gene, based on the chosen FDR.

To identify significant *cis*-eQTL associations, we ran FastQTL in a nominal pass (the default), and defined significant *cis*-eQTLs as those variant-gene pairs whose nominal p-value was below the nominal p-value threshold (at a 5% FDR) for the tested gene.

10.5 Fine-mapping eGene credible sets with SuSiE

To discover the causal SNP(s) driving each *cis*-eQTL signal, we performed fine-mapping with SuSiE[25,79], using the *susieR* package (version 0.12.16) in R. For each tested gene, SuSiE discovers a set of credible causal sets, such that each has some minimum probability of containing a true causal SNP (termed the "coverage" probability), each credible set is made as small as possible, and SNPs within each credible set have some minimum correlation with each other. As such, SuSiE can discover multiple independent signals per gene, and at high resolution.

For each *cis*-eGene identified at a 5% FDR with FastQTL (see section **10.4**), we identified SuSiE credible sets using the following procedure. For each gene, we limit the analysis to the same set of SNPs used with FastQTL, specifically SNPs within 1 Mbp up- and down-stream of the gene's TSS, and with MAF > 0.01. We then remove the effects of the eQTL-mapping covariates (sex, top 5 genotype PCs, 60 PEER factors) from the inverse normal transformed TMM values and genotypes, using the procedure described in this article: https://stephenslab.github.io/susieR/articles/finemapping.html#a-note-on-covariate-adjustment. Finally, we run the `susie_rss` function on the Z-scores from the FastQTL nominal pass, using an in-sample LD matrix calculated from the covariate adjusted genotypes and gene expression variance estimated from the covariate-adjusted expression values. We set the maximum number of credible sets to be 10 (`L=10`), the minimum coverage probability of each credible set to be 0.95 (`coverage=0.95`), and the minimum absolute correlation between SNPs in a credible set to 0.5 (`min_abs_corr=0.5`).

SuSiE discovered credible sets for 9,807 of the 15,022 autosomal eGenes identified in the FastQTL permutation pass and 236 of the 410 chrX eGenes. For each fine-mapped credible set, we select a single representative "lead" eQTL with the highest PIP within that credible set. We use these lead eQTLs in all downstream analyses to represent putative causal eQTL signals.

10.6 Comparison of fine-mapping resolution in subsets of MAGE

To investigate the relationship between fine-mapping resolution and sample diversity of the discovery set, we repeated our eQTL-mapping pipeline for three equally sized (n = 142) subsets of the MAGE data set: one that included only samples in the AFR continental group of 1KGP, a second that included only samples in the EUR continental group of 1KGP, and a third that included samples from all 26 populations of 1KGP. Within each subset, samples were selected from the populations included in the subset as evenly as possible. For each subset, we independently repeated the entire eQTL mapping pipeline (sections **10.1-10.5**) across autosomal genes as before with two minor changes, both related to the smaller size of the sample: 1) the MAF cutoff for eQTL mapping was set to 0.05 rather than 0.01 and 2) only 15 PEER factors were calculated and included instead of 60.

We compared the size of the resulting SuSiE credible sets 1) for genes with at least one SuSiE credible set in any of the subsets (**Fig. S11A**), and 2) for genes with at least one SuSiE credible set in all three subsets (**Fig. S11B**). In both cases, we observe the best resolution (fewest variants per credible set) on average in the African subset, the second-best resolution in the diverse subset, and the worst resolution in the European subset. This result is expected given the increased genetic diversity in African populations[22,23] and highlights the advantages that inclusion of diverse samples affords for detection of causal signals.

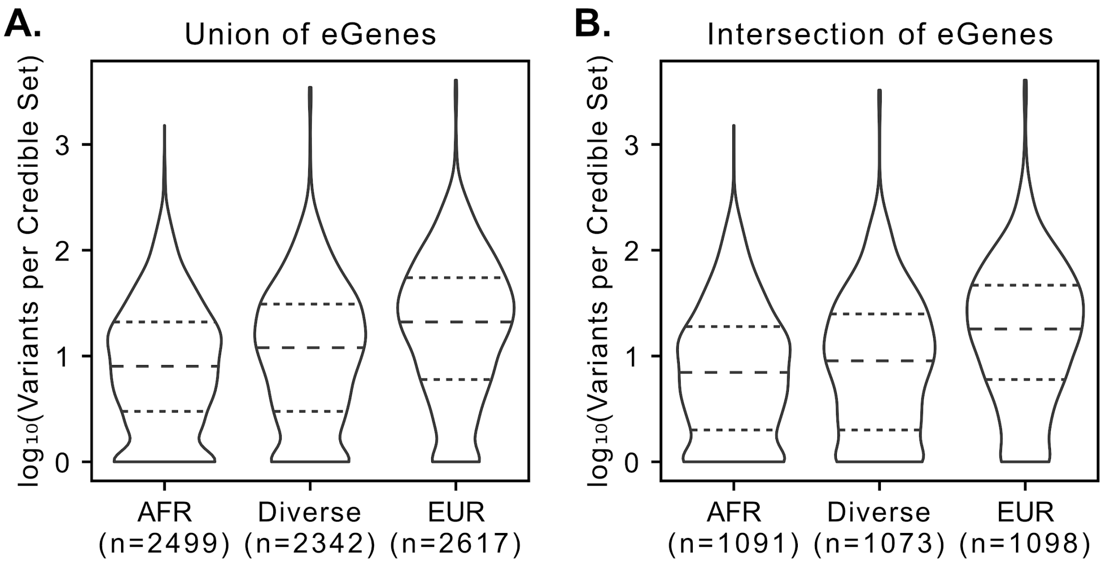

Figure S11. Comparison of fine-mapping resolution in subsets of MAGE. We re-ran the entire eQTL mapping pipeline (including filtering of variants and genes) for three equally sized (n = 142) subsets of the MAGE data set, one that included only samples in the AFR continental group of 1KGP, a second diverse subset that included samples from all 26 populations of 1KGP, and a third that included only samples in the EUR continental group of 1KGP. The pipeline was run separately for each subset. **(A)** The number of variants per SuSiE credible set within each subset for all autosomal genes that had at least one credible set in at least one subset. **(B)** Same as panel A, but only for those genes that had at least one credible set in all three subsets. The AFR subset yields the smallest (best resolution) credible sets, as expected given the increased genetic diversity in African populations[22,23]. Importantly, the diverse subset yields only slightly lower resolution credible sets. The worst resolution is observed in the EUR subset. For both panels, sample sizes describe the number of credible sets in each subset.

10.7 Calculation of Allelic Fold Change (aFC)

While valuable for identifying significant eQTLs, the slope of the regression from the FastQTL nominal pass (see section **10.4**) is based on rank-normalized expression quantifications and as such does not have a clear biological interpretation. At the same time, for eGenes with multiple independent causal signals, the measured "nominal" effect size of one causal eQTL can be influenced by the effects of the other causal signals. As such, for each eGene, it is useful to calculate the "marginal" effect size of each causal signal, conditional on the effects of the other causal signals for that gene. This better reflects the actual effect each causal signal has on the expression of its eGene.

One metric for quantifying the effect of an eQTL on the expression of its eGene is allelic fold change (aFC), which describes the ratio between the expression of the haplotype carrying the alternative allele to the one carrying the reference allele[28]. This concept can be extended to handle multiple causal signals per gene, as implemented in the aFC-n tool[80]. We calculated effect sizes for each lead eQTL in our fine-mapping results (see section **10.5**), limiting the analysis to autosomal genes and genes in the X chromosome PARs, because at the time of publication, aFC-n does not accommodate haploid genotypes in the non-PARs. We calculated the effect size of each variant as log2(aFC) using the following procedure:

First, we generated corrected expression counts for each gene using DESeq2 (version 1.36.0; [71]) in R. Using the salmon-generated pseudocount expression data per sample (detailed in section 4.1), transcript-level counts were first converted into gene-level counts using the tximport (version 1.24.0; [65]) in R under default parameters. These gene-level expression estimates were imported into the DESeq2 ecosystem, and we generated "corrected" expression counts, using the `counts` function with `normalized=TRUE`. These corrected counts are functionally equivalent to read counts but have been corrected for library size and average transcript length. These corrected counts were then log2-transformed (with a +1 pseudocount).

Next, we removed the effects of covariates using the following procedure: for each eGene we fit a linear model that regresses $\log_2$(corrected counts) onto sample genotypes for each lead eQTL of that gene as well as the eQTL-mapping covariates described in section **10.4**. Any covariates whose 95% confidence interval did not include 0 were regressed out from the $\log_2$(corrected counts).

Finally, we used `afcn.py` with the `--conf` option, using these covariate-adjusted $\log_2$(corrected counts) as input, to calculate $\log_2$(aFC) for each lead eQTL (**Extended Data Fig. 1**). We used a slightly modified version of aFC-n version 1.0.0, available here: https://github.com/dtaylo95/aFCn.

11 *cis*-sQTL mapping

11.1 Splicing normalization

Intron-excision ratios from Leafcutter were filtered as described in section **5.2**. Prior to removing splicing clusters with a single intron (but after removing low complexity introns), intron excision ratios were normalized using the Leafcutter `prepare_phenotype_table.py` companion script. We then filtered out splicing clusters with only a single intron. Splicing clusters were mapped to annotated genes in GENCODE v38[63] using the Leafcutter `map_clusters_to_genes.R` companion script. Of the 32,867 splicing clusters remaining after filtering, we removed 679 additional clusters that did not map to annotated exons in GENCODE v38.

For sQTL mapping, normalized intron excision ratios (across all samples) were collected into a bed file, with each cluster annotated with the TSS of the gene to which it mapped. If a cluster mapped to multiple genes, each mapping was included in the bed file separately.

11.2 Calculation of PEER covariates

For sQTL mapping, PEER factors were calculated from the normalized intron excision ratios described in section **11.1**. Previous optimizations performed by GTEx found that performing sQTL mapping with 15 PEER factors as covariates maximized the number of sGenes discovered[26]. To ensure that this was an appropriate selection for MAGE, we performed sQTL mapping (as described in section **11.3**, below) on Chromosome 1 with 0, 1, 2, 5, 10, 15, 20, 25, 30, 40, or 50 PEER factors included as covariates (**Fig. S9**). We observed that the number of sGenes discovered is relatively robust to the number of PEER factors included as covariates. For consistency with GTEx, we therefore chose to use 15 PEER factors as covariates when performing sQTL mapping.

As with the expression-derived PEER factors, we observed that these PEER factors correlate strongly with sequencing batch and more weakly with sample continental group and population (**Fig. S10C,D**). Because of their correlation with continental group and population, we elected not to include these factors as covariates for analyses of differential splicing between continental groups/populations (section **9**; **Fig. 2C,D**).

11.3 Discovery of nominal *cis*-sQTLs with FastQTL

The *cis*-sQTL mapping procedure largely matched the procedure used to map *cis*-eQTLs, as described in section **10.4**. For each of the 11,912 autosomal genes and 388 X chromosome genes with splicing clusters that passed filtering thresholds (see section **11.1**), we regressed normalized intron excision ratios onto variant genotypes for all variants within 1 Mbp up- and down-stream of the gene's transcription start site (TSS), and with MAF > 0.01. The top 5 genotype PCs (section **10.2**), 15 PEER factors (section **11.2**), and sample sex were included as covariates. For variants in X chromosome non-PARs, the haploid genotypes of XY samples were transformed as described in section **10.4**.

As with *cis*-eQTL mapping, we first ran FastQTL in the adaptive permutation mode using `--permute 1000 10000` to discover significant *cis*-sGenes (genes with at least one *cis*-sQTL). We used grouped permutations (using the `--`

`phenotype_groups` option) to compute a gene-level empirical p-value over all splicing clusters of a gene. We discovered *cis*-sGenes and calculated per-gene nominal p-value thresholds at a 5% FDR (as described in section **10.4**).

To identify significant *cis*-sQTL associations, we ran FastQTL in a nominal pass and defined significant *cis*-sQTLs as those variant-intron pairs whose nominal p-value was below the 5% FDR nominal p-value threshold for the tested gene.

11.4 Fine-mapping sGene credible sets with SuSiE

For each 5% FDR *cis*-sGene identified, fine-mapping was performed separately for each intron mapping to that gene (termed sIntrons; limited to only the introns that passed filtering). Fine-mapping was done as described for *cis*-eGenes (section **10.5**), mapping normalized intron excision ratios onto sample genotypes, using the same set of covariates used for sQTL mapping with FastQTL. All other options remained the same.

SuSiE discovered credible sets (for at least one intron) for 6,604 of the 7,727 autosomal sGenes identified in the FastQTL permutation pass. Of the 25,864 fine-mapped sIntrons, 4,425 (17%) had more than one credible set (**Extended Data Fig. 2A**), representing 1,777 (27%) of the 6,604 fine-mapped sGenes. Of the 32,438 intron-level credible sets, 7,720 (24%) contained just a single variant (median 6 variants per credible set; mean = 22.4; s.d. = 114.4; **Extended Data Fig. 2B**).

On the X chromosome, SuSiE discovered credible sets (for at least one intron) for 135 of the 146 sGenes identified in the FastQTL permutation pass. Of the 510 fine-mapped sIntrons, 88 (17%) had more than one credible set, representing 30 (22%) of the 135 fine-mapped sGenes. Of the 633 intron-level credible sets, 232 (37%) contained just a single variant (median 3 variants per credible set; mean = 15.7; s.d. = 42.4).

To obtain a gene-level summary of the sQTL fine-mapping results, we collapsed these intron-level credible sets into gene-level credible sets. For each sGene, we iteratively merged all intron-level credible sets that overlapped by at least one variant. The result is a set of gene-level merged credible sets that are independent from one another (no variants in common) and whose union is equivalent to the union of the input intron-level credible sets. Of the 6,604 fine-mapped autosomal sGenes, 3,490 (53%) had more than one credible set (**Extended Data Fig. 2C**). Of the 16,451 gene-level credible sets, 3,569 (22%) contained just a single variant (median 7 variants per credible set; mean = 23.6; s.d. = 99.1; **Extended Data Fig. 2D**). On the X chromosome, of the 284 gene-level credible sets, 80 (28%) contained just a single variant (median 5 variants per credible set; mean = 21.7; s.d. = 53.0).

Analogous to selection of lead eQTLs described in section **10.5**, we selected a representative "lead" sQTL for each gene-level merged credible set by first determining the intron-level credible set with the greatest coverage among those comprising the gene-level merged credible set, and then selecting the variant within that intron-level credible set with the highest PIP. We use these lead sQTLs in all downstream analyses to represent putative causal sQTL signals.

12 Analysis of negative selection

Evidence of negative selection on regulatory variation affecting highly constrained genes was assessed by intersecting our eQTL fine-mapping results with orthogonal gene-level metrics of constraint generated in previous studies based on depletion of loss of function point mutations or copy number variation[30,81–85] (**Extended Data Fig. 3A**). We additionally assessed evidence of negative selection within putative promoter elements, defined based on various intervals around the TSS ([-1000, 1000] bp, [-500, 0] bp, [-50, 0] bp; **Extended Data Fig. 3B**). To avoid circularity in the latter case, evidence of negative selection within promoters was assessed based on levels of sequence conservation across species (as opposed to polymorphism within human populations), based on mean PhyloP[86] scores obtained from Cactus[87] alignments of 447 mammal species, including Zoonomia genomes[88]. In both cases, we restricted our analysis to protein-coding autosomal genes exceeding the minimum expression threshold used in our differential expression and eQTL mapping pipelines. Constraint metrics obtained from gnomAD[82,85] were restricted to MANE Select[89] transcripts to avoid double-counting of genes with multiple isoforms. Among this set of genes, we identified the top 10th percentile of highly constrained genes

based on each constraint metric, accounting for their differences in directionality (e.g., high pLI scores but low RVIS scores denote evidence of constraint). The remaining 90% of genes were considered as the background for comparison. We then contrasted the number of independent credible causal sets per gene between the constrained and background set using a quasi-Poisson generalized linear model where normalized mean expression level (i.e., `baseMean`, as computed with DESeq2[71]) was included as a continuous numerical covariate. We also compared the distributions of effect sizes of the lead eQTL per credible causal set between constrained and background sets of genes using a two-tailed Mann–Whitney U test, where the base-2 logarithm of the the absolute value of the estimated allelic fold change ($|\log_2(\text{aFC})|$) was used as input to each model, as computed with aFC-n[80].

13 Functional annotation and enrichment of fine-mapped *cis*-QTLs

13.1 Functional annotation of *cis*-eQTLs

We performed a functional enrichment analysis in order to evaluate the association between fine-mapped *cis*-eQTLs and transcription factor (TF) as well as chromatin regulator (CR) binding sites. The data for this analysis was obtained from the ENCODE Project Consortium, specifically the ENCODE regulation track transcription factor binding site cluster ChIP-seq index file, which encompasses information for 338 DNA-binding proteins across 129 cell types[33,90]. We employed GenomicsRanges (version 1.38.0)[91] and BEDTools (version 2.29.2)[92] to intersect *cis*-eQTL variants with TF binding sites. Our subsequent enrichment tests were performed using the GREGOR (version 1.3.1) Perl-based pipeline[93]. At a high level, this involves summing the binomial random variables corresponding to the count of index SNPs located within any given TF feature, followed by the computation of enrichment p-values via saddlepoint approximation.

We defined the criterion for positional overlap between SNPs and regulatory features as a minimum of one base pair (≥1 bp) intersection. The fold enrichment for each transcription factor binding site was then calculated as a ratio, defined as the observed fraction of index SNPs overlapping the TF binding sites divided by the expected mean overlap with a matched control SNP set. The control SNPs were matched by the index SNPs' minor allele frequencies and their proximity to the nearest gene's transcription start site (TSS distance), thereby providing a robust basis for comparison ensuring that any observed enrichment is not due to underlying biases in SNP distribution with respect to allele frequency or genomic location. Statistical significance for enrichment was assessed against a background distribution of matched control SNPs, with Bonferroni correction for multiple hypothesis testing to control the family-wise error rate. The results of this analysis are shown in **Fig. S12A**.

To assess chromatin association of the lead eQTLs, we quantified the enrichment of autosomal lead eQTLs within the core 15-predicted chromatin states from the Roadmap Epigenomics Consortium[32], which was produced using ChromHMM v1.10[94], based on a multivariate hidden Markov model. The model delineates the genome into 15 distinct chromatin states based on the combined presence of five key histone modifications: H3K4me3, H3K4me1, H3K36me3, H3K27me3, and H3K9me3. To evaluate enrichment, we examined all 127 reference epigenomes from the Roadmap Epigenomics Consortium encompassing diverse cell types and tissues to ensure a broad representation of epigenetic landscapes, for which we utilized consolidated narrowPeak files for each of 127 epigenomic mappings (**Fig. S12B**). To further parse cell type-specific patterns and consider the predicted enrichment across cell/tissue types, we quantified the enrichment in primary DNAse Hypersensitivity Sites (DHS) data across a diverse panel of 53 cell and tissue types provided by the Roadmap Epigenomics Consortium (**Fig. S12C**).

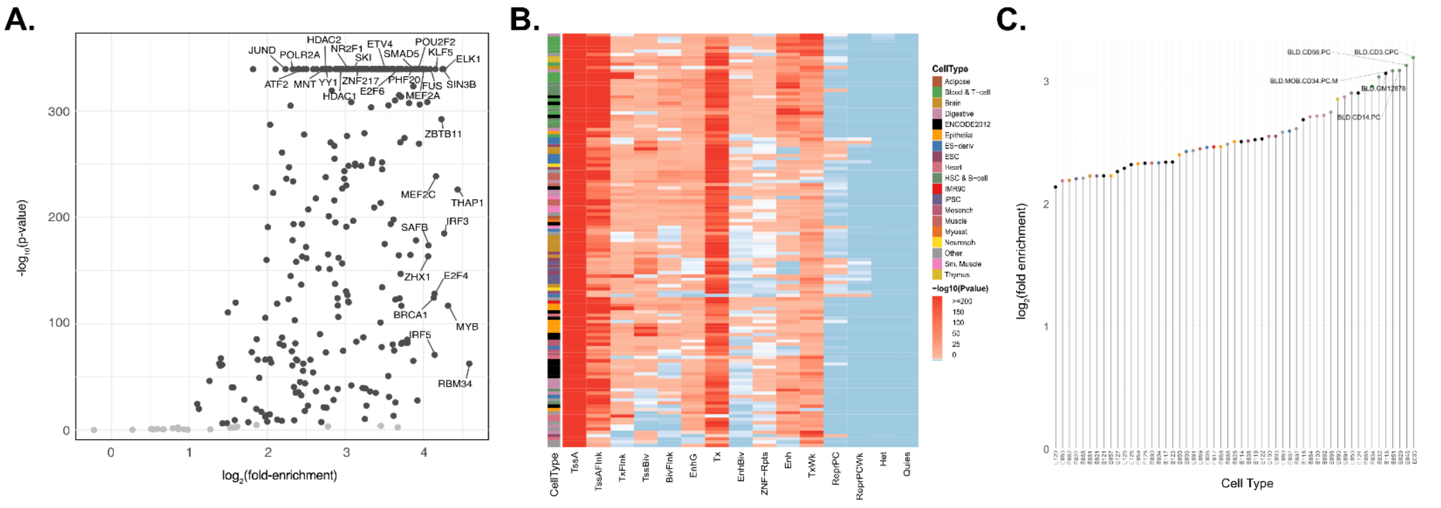

Figure S12. Lead eQTLs are enriched within regulatory elements across cell and tissue types. (A) Enrichment analysis of lead cis-eQTLs (n=14,963 unique eVariants) at TFBS (Transcription Factor Binding Sites) from ENCODE ChIP-seq binding profiles. Data points with p-values < 0.001 (Bonferroni corrected) and log2(fold-enrichment) > 1 are colored in black. **(B)** Corresponding heatmap to **Fig. 4A** showing significance of the enrichment estimates (right tailed, binomial P-value). Differential eQTL enrichment across various chromatin states in multiple cell types, highlighting pronounced enrichment at active transcription start sites (TssA) and proximal flanking regions (TssAFlnk), with moderate enrichment in enhancer regions (Enh, EnhG), particularly in blood cell types. In contrast, regions characterized by quiescence, repression, and heterochromatin show a marked depletion of eQTLs. **(C)** A lollipop plot showing the pronounced enrichment of lead eQTLs in DNAse hypersensitivity sites (DHSs) across 53 cell/tissue types (colored as in A). We note a marked enrichment in DHS of blood cell types, with lymphoblastoid cell line GM12878 appearing as one of the top hits.

We also assessed the distribution of eQTL effect sizes across all 15 predicted chromatin states as annotated with ChromHMM by the Roadmap Epigenomics Consortium. The effect sizes were quantified as the base-2 logarithm of the the absolute value of the estimated allelic fold change (|$\log_2$(aFC)|) across 15 different chromatin states specific to LCLs. Next, to elucidate the regulatory potential of lead *cis*-eQTLs, we assessed the distribution of their effect sizes across promoter, enhancer, and dyadic regions in LCLs associated to multi-tissue DHS data to ensure a comprehensive evaluation of active chromatin domains. Median cis-eQTL effect sizes were compared across these regions to discern any preferential associations. We further stratified eQTLs by effect size, delineated into deciles of absolute $\log_2$ allelic fold change (|$\log_2$(aFC)|), and analyzed their enrichment within the chromatin states predicted for LCLs, including other primary blood cell types. Critically, we also examined how these patterns generalize to other primary blood cell types, including Primary B-cells, T-cells, Natural Killer Cells, and Hematopoietic Stem Cells (**Fig. S13-S17**).

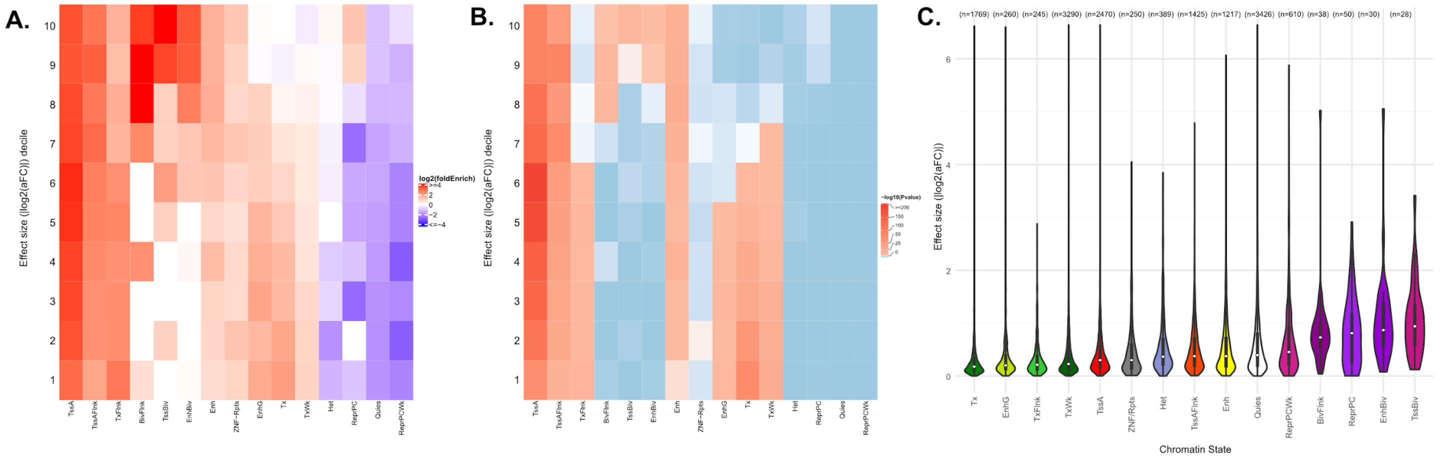

Figure S13. Lead eQTLs are enriched within regulatory regions of lymphoblastoid cell line GM12878 (E116). (A) Enrichment analysis of the decile partitioned eQTL effect sizes (|$\log_2$(aFC)|) across 15 different chromatin states predicted by chromHMM model specific to LCLs. **(B)** Corresponding heatmap to panel A, showing significance of the decile-based enrichment analysis estimates (right tailed, binomial P-value). We

observed consistent promoter-associated enrichment across deciles. Conversely, significant enrichment peaks in bivalent regions (TssBiv, EnhBiv, BivFlnk) are specifically observed among eQTLs with the largest effect sizes. **(C)** Distribution of effect sizes for lead eQTLs within Roadmap Epigenomics chromHMM predicted chromatin states[32] exhibiting a trend of diminished effect sizes for transcribed regions (Tx, TxWk, and TxFlnk).

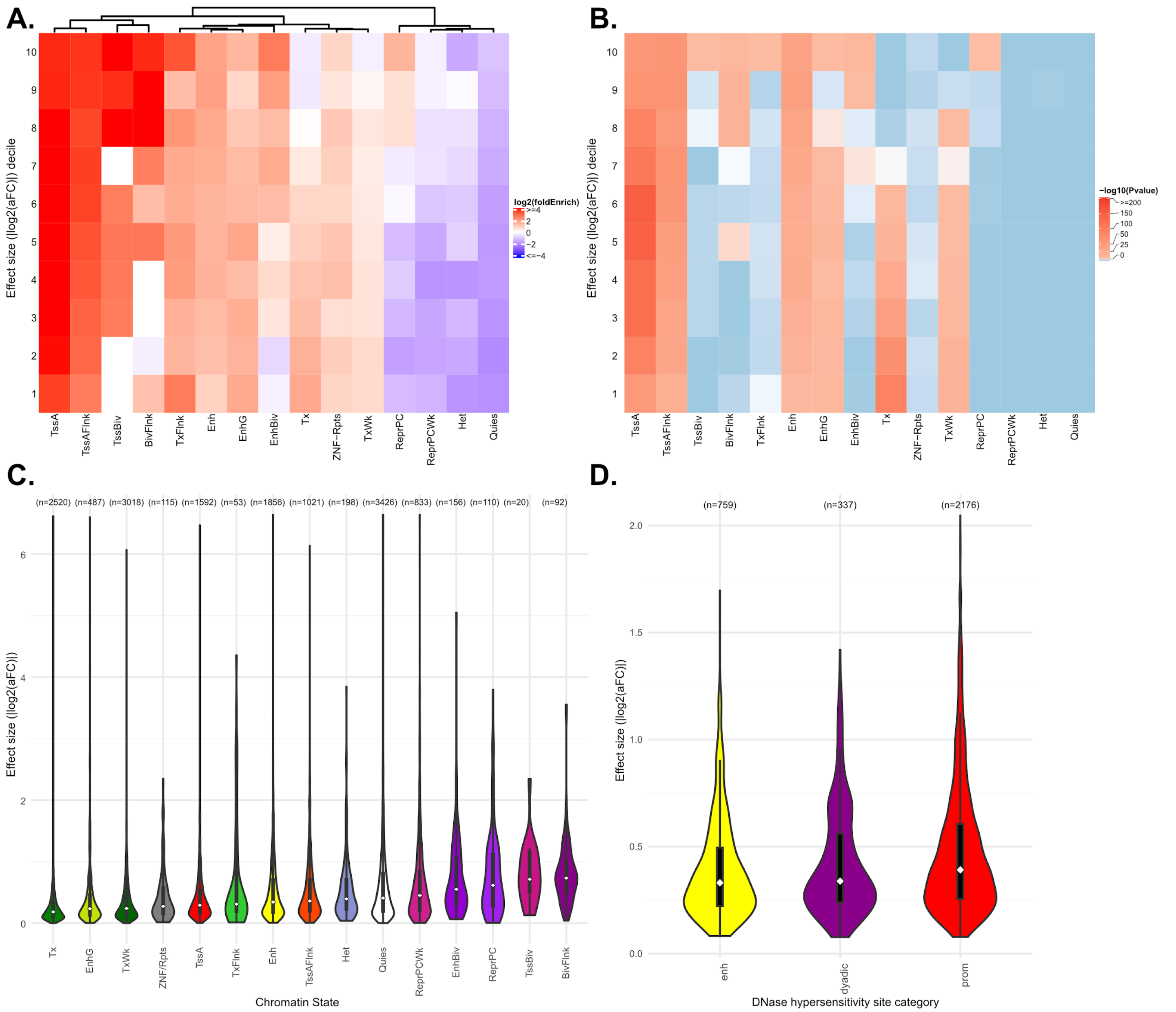

Figure S14. Lead eQTLs are enriched within regulatory regions of primary B-cells (E032). (A) Decile-based enrichment analysis of lead eQTL effect sizes measured as absolute value of the estimated allelic fold change ($|\log_2(\text{aFC})|$) across 15 different chromatin states predicted by the Roadmap Epigenomics chromHMM model[32] specific to Primary B-Cells. Heatmap showcases promoter associated regions maintaining a consistent trend across all deciles of eQTL effect sizes. In contrast, a notable enrichment is detected within bivalent regions, Bivalent Transcription Start Sites (TSSBiv), Bivalent Enhancers (EnhBiv) and Bivalent flanking regions (BivFlnk), which is most pronounced for eQTLs demonstrating large effects. **(B)** Corresponding heatmap to panel A, showing significance of the enrichment estimates (right tailed, binomial P-value). **(C)** Distribution of effect sizes for lead eQTLs within chromatin states exhibiting pronounced trend of diminished effect sizes for transcribed regions (Tx, TxWk, and TxFlnk) **(D)** Distribution of absolute effect sizes of lead eQTLs (measured as $\log_2(\text{aFC})$) across chromatin states in Primary B-Cells that are associated with multi-tissue DNAse hypersensitivity sites[32].

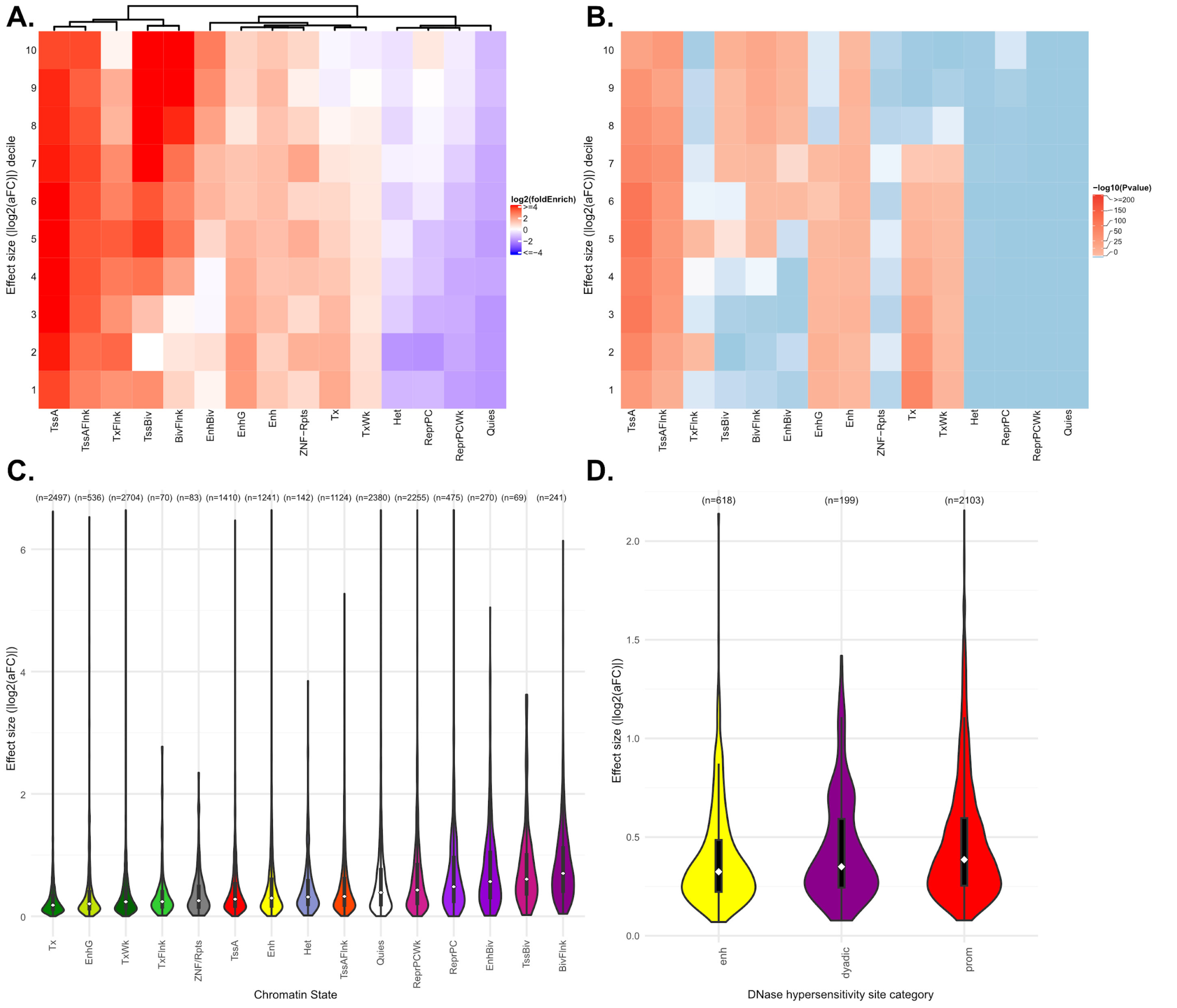

Figure S15. Lead eQTLs are enriched within regulatory regions of primary T-cells (E034). (A) Decile-based enrichment analysis of lead eQTL effect sizes measured as absolute value of the estimated allelic fold change (|$\log_2$(aFC)|) across 15 different chromatin states predicted by chromHMM model specific to Primary T-Cells . Heatmap showcases promoter associated regions maintaining a consistent trend across all deciles of eQTL effect sizes. In contrast, a notable enrichment is detected within bivalent regions, Bivalent Transcription Start Sites (TSSBiv), Bivalent Enhancers (EnhBiv) and Bivalent flanking regions (BivFlnk), which is most pronounced for eQTLs demonstrating large effects. **(B)** Corresponding heatmap to panel A, showing significance of the enrichment estimates (right tailed, binomial P-value). **(C)** Distribution of effect sizes for lead eQTLs within chromatin states exhibiting pronounced trend of diminished effect sizes for transcribed regions (Tx, TxWk, and TxFlnk) **(D)** Distribution of absolute effect sizes of lead eQTLs measured as $\log_2$(aFC) across chromatin states in Primary T-Cells that are associated with multi-tissue DNAse Hypersensitivity Sites[32].

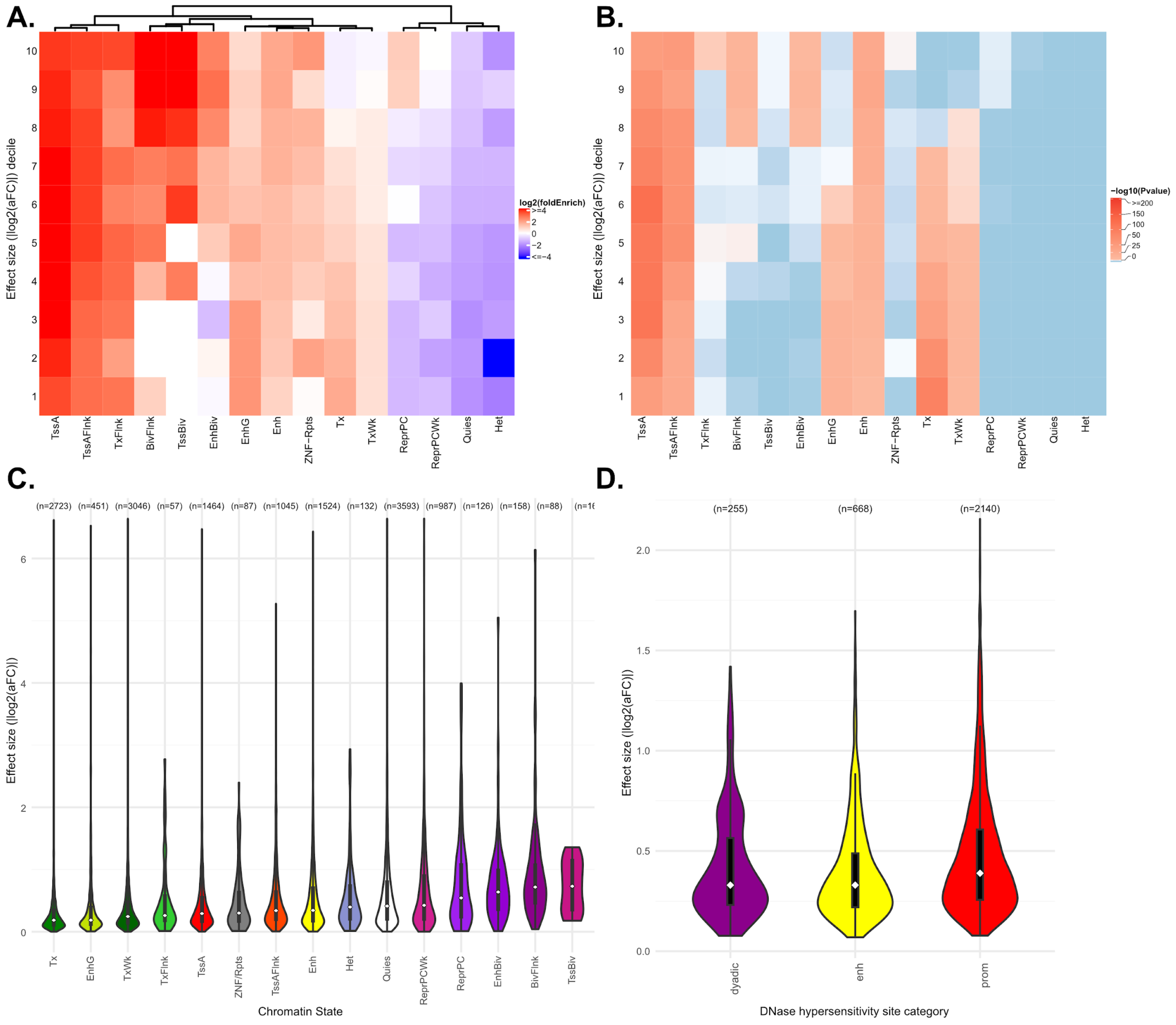

Figure S16. Lead eQTLs are enriched within regulatory regions of primary natural killer cells (E046). (A) Decile-based enrichment analysis of lead eQTL effect sizes measured as absolute value of the estimated allelic fold change ($|\log_2(\text{aFC})|$) across 15 different chromatin states predicted by chromHMM model specific to Primary Natural Killer Cells. Heatmap showcases promoter associated regions maintaining a consistent trend across all deciles of eQTL effect sizes. In contrast, a notable enrichment is detected within bivalent regions, Bivalent Enhancers (EnhBiv) and Bivalent flanking regions (BivFlnk), which is most pronounced for eQTLs demonstrating large effects. **(B)** Corresponding heatmap to panel A, showing significance of the enrichment estimates (right tailed, binomial P-value). **(C)** Distribution of effect sizes for lead eQTLs within chromatin states exhibiting pronounced trend of diminished effect sizes for transcribed regions (Tx, TxWk, and TxFlnk) **(D)** Distribution of absolute effect sizes of lead eQTLs measured as $\log_2(\text{aFC})$ across chromatin states in Primary Natural Killer Cells that are associated with multi-tissue DNAse Hypersensitivity Sites[32].

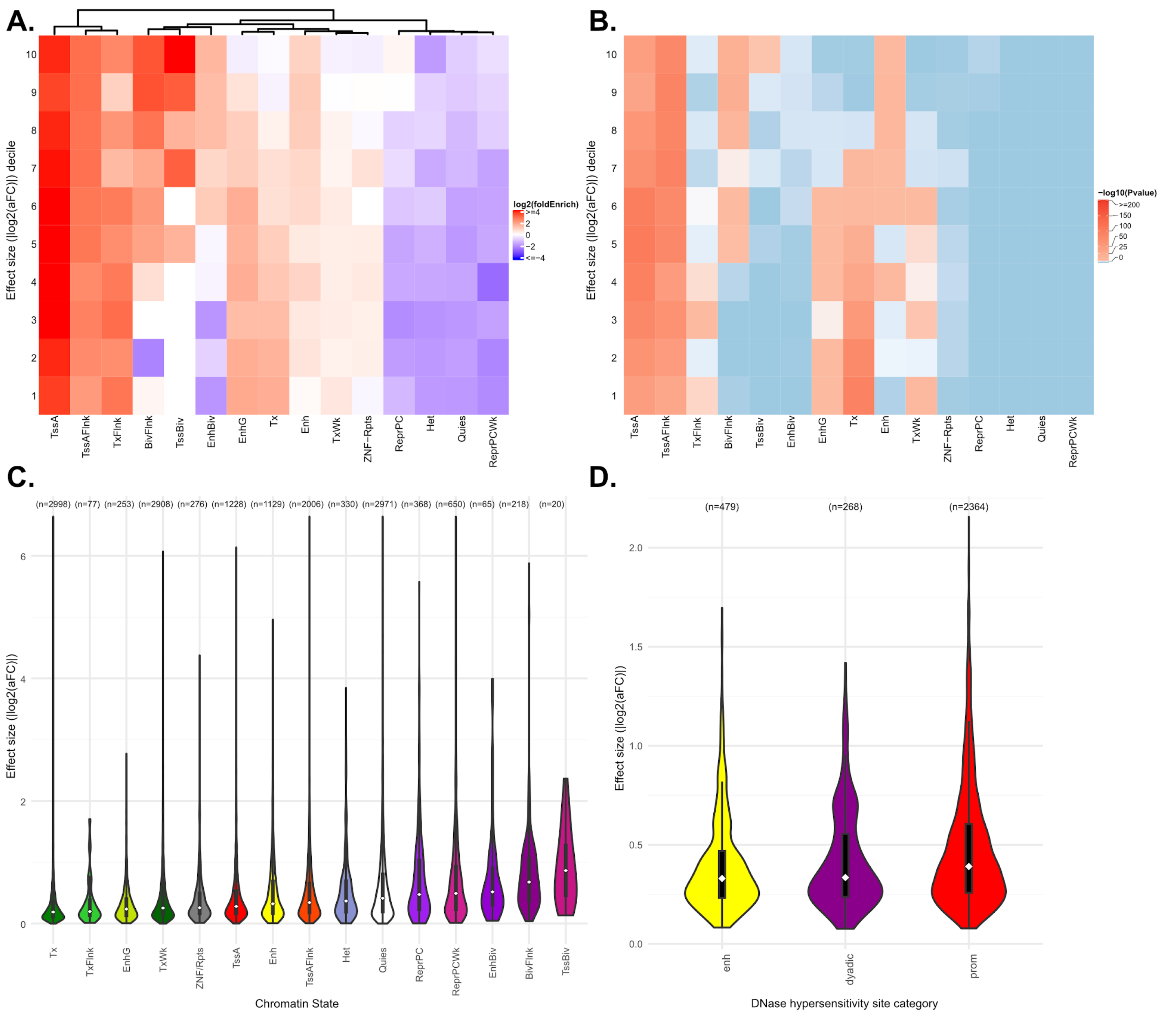

Figure S17. Lead eQTLs are enriched within regulatory regions of primary hematopoietic stem cells (E051). (A) Decile-based enrichment analysis of lead eQTL effect sizes measured as absolute value of the estimated allelic fold change ($|log_2(aFC)|$) across 15 different chromatin states predicted by chromHMM model specific to Primary Hematopoietic Stem Cells. Heatmap showcases promoter associated regions maintaining a consistent trend across all deciles of eQTL effect sizes. In contrast, a notable enrichment is detected within bivalent regions, Bivalent Transcription Start Sites (TSSBiv) and Bivalent flanking regions (BivFlnk), which is most pronounced for eQTLs demonstrating large effects. **(B)** Corresponding heatmap to panel A, showing significance of the enrichment estimates (right tailed, binomial P-value). **(C)** Distribution of effect sizes for lead eQTLs within chromatin states exhibiting pronounced trend of diminished effect sizes for transcribed regions (Tx, TxWk, and TxFlnk) **(D)** Distribution of absolute effect sizes of lead eQTLs measured as $log_2(aFC)$ across chromatin states in Primary Hematopoietic Stem Cells that are associated with multi-tissue DNAse Hypersensitivity Sites[32].

The enrichment calculations for both chromatin states and DHS peaks were conducted using the same GREGOR Perl script pipeline[93], as previously applied in the transcription factor binding site enrichment analysis. The enrichment was quantified using log_2 fold changes (observed/expected) and p-values ($-log_{10}$ transformed) to determine the magnitude and significance of enrichment across chromatin states and DHS sites across 127 cell/tissue samples.

13.2 Functional annotation of *cis*-sQTLs

To assess the genomic context of fine-mapped *cis*-sQTLs, we measured the enrichment of lead sQTLs in genomic annotations from the Variant Effect Predictor (VEP) tool (version 109)[95]. The VEP tool annotates variants with 31 non-mutually-exclusive genomic annotations, including splicing-related regions, intergenic regions, introns, non-coding transcripts, and transcription factor (TF) binding sites. We excluded any annotations from our analysis for which fewer than five lead sQTLs were annotated, resulting in a final set of 26 genomic annotations. Additionally, to further contextualize the functional impact of sQTLs in MAGE, we used the Loss-of-Function Transcript Effect Estimator (LOFTEE) tool (version 1.0.2)[82] as a plugin for VEP, which provides loss-of-function (LoF) predictions with high confidence (LoF Hc) and low confidence (LoF Lc) categories. LoF annotations include stop-gained, splice site disrupting, and frameshift variants.

We used VEP and LOFTEE to annotate all lead sQTLs, as well as a matched null set of variants for comparison. The null set of variants was chosen to match the lead sQTLs based on minor allele frequency (MAF) and distance from the transcription start site (TSS). Following annotation, we quantified sQTL fold enrichment in each annotation, defined as the ratio between the proportion of lead sQTLs in the annotation to the proportion of matched null variants in the annotation. A pseudocount of 1 was added to annotations with no annotated matched null variants. To evaluate the significance of enrichment of sQTLs within each annotation, we performed a Fisher's exact test (One-sided test, alternative = "greater"). Statistical significance was determined against this random null background distribution, employing a Bonferroni correction to account for multiple hypothesis testing.

14 Colocalization of *cis*-QTLs with complex trait GWAS

We performed colocalization analysis to discover shared signals between fine-mapped MAGE *cis*-e/sQTLs and GWAS results from the Population Architecture using Genomics and Epidemiology (PAGE) study[8]. Specifically, we evaluated colocalization for 25 traits with PAGE summary statistics available from the NHGRI-EBI GWAS catalog[96] **(Fig. S18).** Harmonized summary statistics reported on the GRCh38 reference were downloaded from the GWAS Catalog FTP server for each trait. The full set of traits included in this analysis is described in **Table S1**.

For each trait, we identified independent significant GWAS signals by iteratively selecting the SNP with the lowest p-value (below a significance threshold of $p < 5 \times 10^{-8}$) and then removing from selection all SNPs within 1 Mbp up- or downstream. This process was repeated until no additional SNPs could be selected. The resulting set of "sentinel variants" represents independent GWAS signals for that trait. Across the 25 traits we included in this analysis, we identified 384 such independent sentinel variants.

For use in the colocalization analysis, we calculated a PAGE LD matrix in a +/- 500 kbp window around each sentinel variant. Using imputed genotypes from TOPMed[97], we subset the data to only the samples for which the corresponding trait was measured and to the region +/- 500 kbp of the sentinel variant, then calculated an LD matrix using the `--keep-allele-order --r square` arguments in PLINK[62].

For each sentinel variant, we performed colocalization with MAGE cis-eQTLs (described in section 10.4) in a +/- 500 kbp window around the sentinel variant. This prevented overlapping tests between sentinel variants. For each sentinel variant-eGene pair, we performed colocalization as follows. We subset the PAGE summary statistics and MAGE nominal eQTL results to this region (also requiring the eGene TSS to be within the region). We used coloc[34,35] with SuSiE to perform colocalization between the PAGE GWAS signal and each eGene in the colocalization window. We subset the analysis to variants with summary statistics and LD information in both datasets. We used the `runsusie()` function from coloc to run SuSiE on the GWAS summary statistics, using the LD matrix described above and with the same SuSiE arguments used for fine-mapping (described in section **10.5**). For the eGene, we used the `runsusie()` function from coloc to run SuSiE on the eQTL nominal pass summary statistics, using covariate-adjusted in-sample LD matrix described in section

10.5 and with the same SuSiE arguments used for fine-mapping. We then use the `coloc.susie()` function from coloc to perform colocalization between the two sets of SuSiE results. This function tests for colocalization between each pair of credible sets produced by the `runsusie()` function between the two datasets and reports the probability that the two credible sets represent the same causal signal. We defined moderate colocalizations as those with posterior probabilities ≥ 0.5 and strong colocalizations as those with posterior probabilities ≥ 0.8. We emphasize that this approach 1) allows for multiple causal signals at each GWAS sentinel variant and for each eGene and 2) allows for different patterns of LD in each of the two datasets.

For each of the 384 PAGE sentinel variants, we repeated this analysis to discover shared signals with MAGE sQTLs. The procedure was identical to the one described above, but we performed colocalization between the GWAS signal and each MAGE sIntron (described in section **11.4**) in the test window.

For all downstream analyses of these colocalization signals, we considered the full set of fine-mapped e/sQTLs from MAGE when interpreting the colocalization signal, not only the subset of variants that were included in the colocalization analysis (variants with summary statistics and LD information in both datasets).

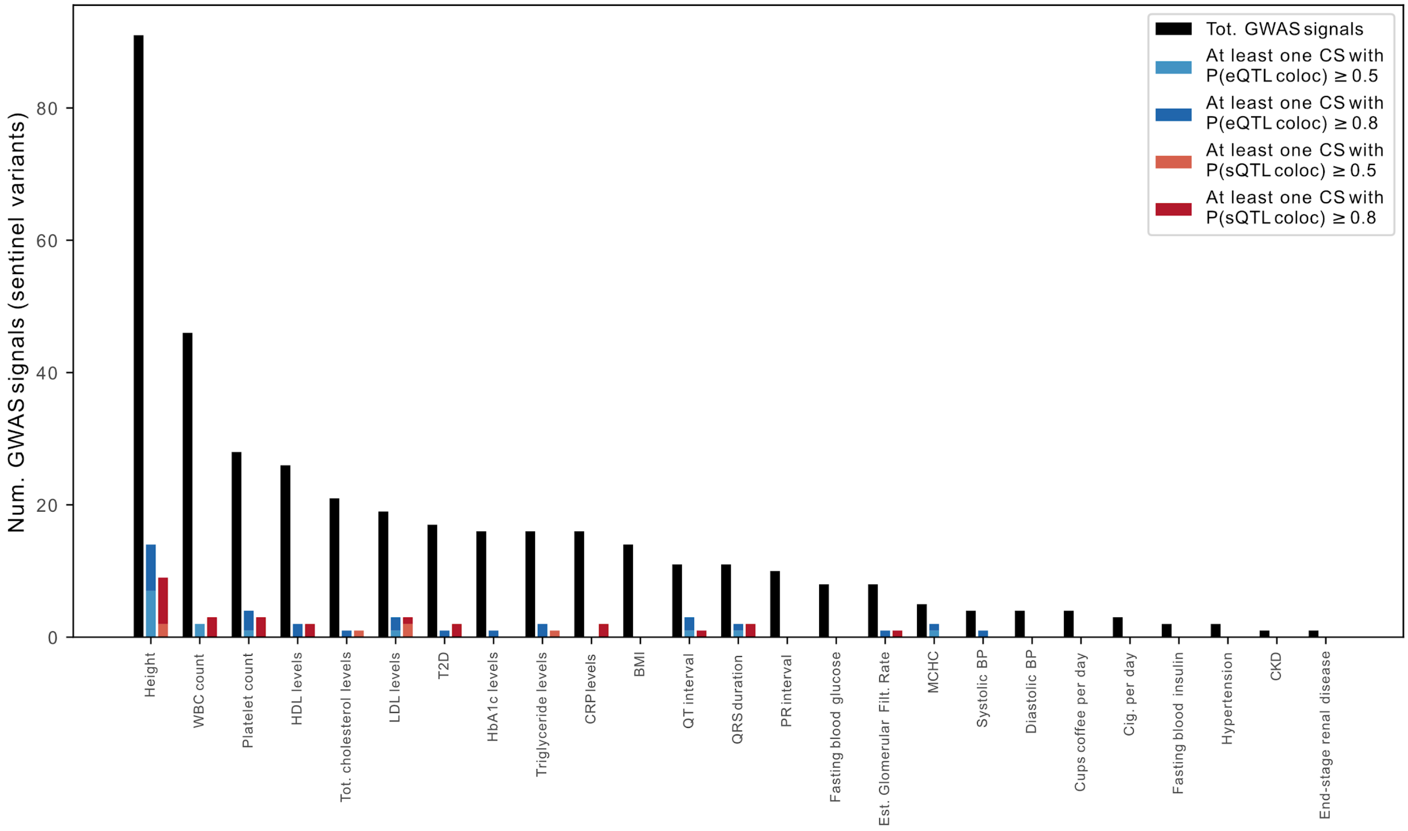

Figure S18. Colocalization of complex trait GWAS hits with MAGE *cis*-e/sQTLs. Colocalization results across 25 complex traits in PAGE are summarized above. The total number of independent significant GWAS signals (sentinel variants) identified for each trait is depicted with black bars. The number of these GWAS signals where at least one credible set showed moderate (P[eQTL coloc] ≥ 0.5) or strong (P[eQTL coloc] ≥ 0.8) evidence of colocalization with a MAGE eQTL credible set is depicted with light blue and dark blue bars, respectively. The number of GWAS signals where at least one credible set for that signal showed moderate (P[sQTL coloc] ≥ 0.5) or strong (P[sQTL coloc] ≥ 0.8) evidence of colocalization with a MAGE sQTL credible set is depicted with light red and dark red bars, respectively. Bars depicting strong colocalization are overlaid on bars depicting moderate colocalization (i.e., individual GWAS signals were not double-counted for strong and moderate evidence of colocalization).

15 Lead e- and sQTL AF differentiation between populations

To visualize the joint frequency spectrum of autosomal lead eQTLs, we used the GeoVar software package (version 1.0.2)[98]. For visualization, we defined the following discrete allele frequency categories for visualization based on the within-data set alternative allele frequency: unobserved (allele frequency = 0%), rare (0% < allele frequency < 5%), and common (allele frequency > 5%). All allele frequencies were calculated using bcftools (version 1.17). We used GeoVar to visualize the joint frequency spectrum of all lead eQTLs (**Extended Data Fig. 4A**), lead eQTLs that are rare or unobserved in at least one continental groups (**Extended Data Fig. 4B**), lead eQTLs that are unobserved in the EUR continental group (**Extended Data Fig. 4C**), and lead eQTLs that are unobserved in both EUR and AFR continental groups (**Extended Data Fig. 4D**).

We also investigated the joint frequency spectrum of gene-level lead sQTLs (**Extended Data Fig. 5**) and observed qualitatively similar patterns.

16 Replication of credible sets in GTEx

16.1 Defining replicating vs. non-replicating eQTLs

To appropriately compare *cis*-eQTL signals between MAGE and GTEx[26], we first collapsed tissue-specific DAP-G credible sets from GTEx into cross-tissue merged credible sets (ctmCS) for each autosomal gene. To construct the ctmCS, for each gene, we combined the fine-mapping credible sets inferred using DAP-G across all tissues for that specific gene (restricting to variants with PIP > 0.95)[99]. To combine the per-tissue credible sets we iteratively joined any DAP-G credible sets sharing variants, resulting in a set of non-overlapping variants per ctmCS per gene. We considered a MAGE credible set to replicate in GTEx if any variant contained in the MAGE credible set was also contained in at least one GTEx ctmCS for that gene. To define the lead eQTL within each GTEx ctmCS, we first select the tissue-level credible set with the highest coverage amongst those that comprise the ctmCS, and from that tissue-level credible set, we select the variant with the highest PIP. We observe that the set of GTEx ctmCS's that do not replicate in MAGE is enriched for ctmCS's that comprise tissue-level CS's from only a single tissue (hence are tissue-specific; **Extended Data Fig. 6**).

16.2 Functional annotation and enrichment of non-replicating eQTLs

Because so many of the MAGE lead eQTLs did not replicate in the GTEx fine-mapping results, we were acutely curious whether this subset of our results was enriched for functional variation. To address this question, we repeated the analysis described in section **13** for the subset of MAGE eQTLs that did not replicate in the GTEx DAP-G results. Briefly, to evaluate chromatin context of MAGE specific eQTLs, we performed the enrichment and functional annotation analysis across all the 15 predicted chromatin states in 127 epigenomic mappings and 53 primary DHS data from Roadmap Epigenomics as described in section **13**. Our findings mirrored previous results, demonstrating similar functional enrichment patterns between the non-replicating subset of MAGE eQTLs and the full set of results (**Fig. S19A-C**). Additionally, to examine the relationship between fine-mapped MAGE-specific lead eQTLs and the binding sites of transcription factors (TFs) and chromatin regulators (CRs), we conducted a detailed enrichment analysis with 338 DNA associated ChIP-seq profiles obtained from the ENCODE Project Consortium as described in section **13**. As with the full set of lead eQTLs, we observed that MAGE-specific lead eQTLs were highly enriched in TF binding site annotations (**Fig. S19D**).

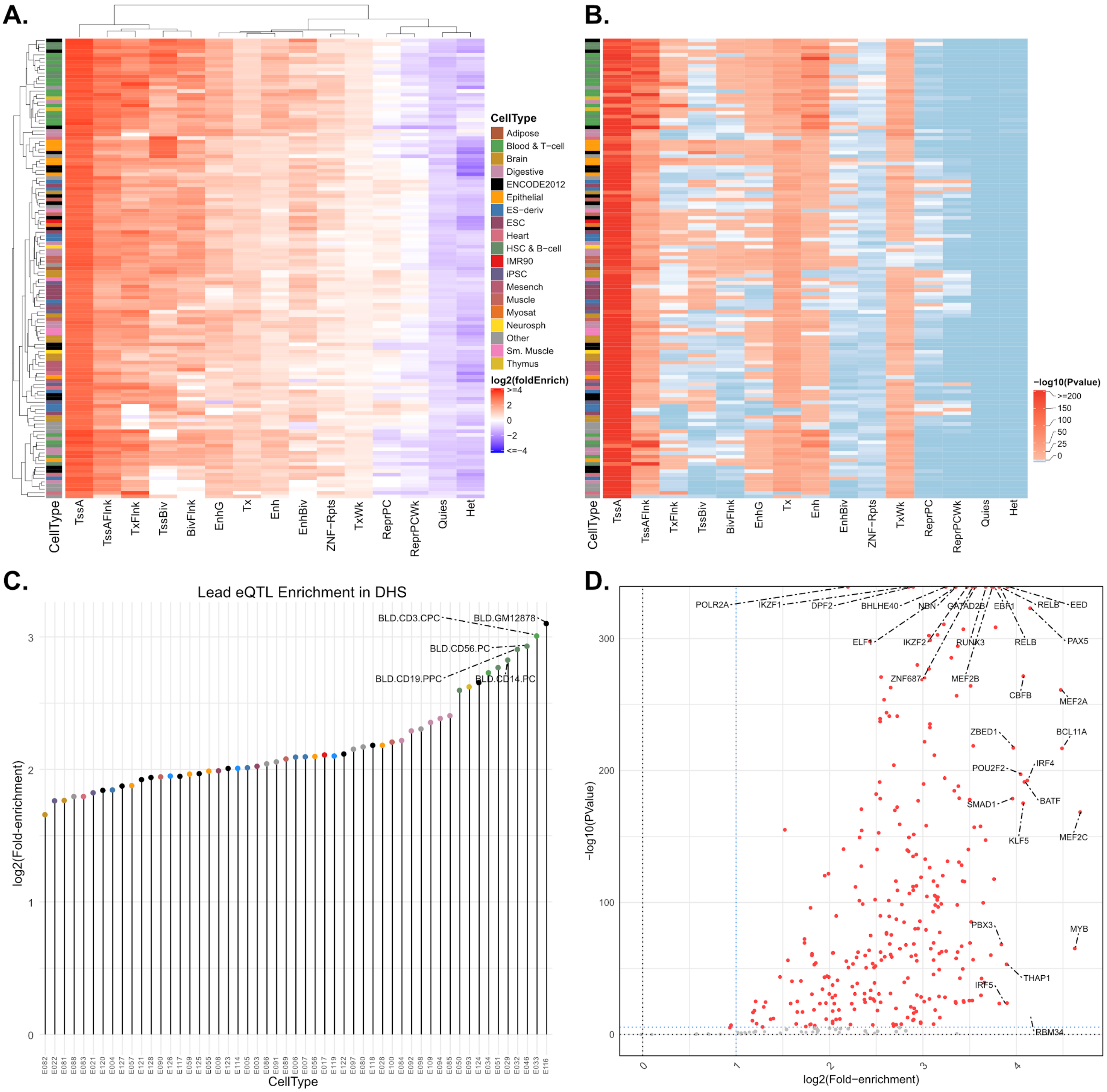

Figure S19. Lead eQTLs that did not replicate in GTEx are functionally enriched in the regulatory regions of pertinent cell types. (A) A heatmap showing the enrichment of unique lead eQTLs at 15 predicted chromatin states across 127 cell/tissue types from the Roadmap Epigenomic Consortium. Cell type annotations are displayed by colored legend keys. Lead eQTLs exhibited strong enrichment at promoter (TssA, TssAFlnk) and enhancer regions (Enh, EnhG) both, with promoter regions showing more pronounced enrichment compared to enhancer regions across the cell types. Strong Hierarchical clustering of Blood and T cells highlighted by green colored cell type annotation bar on the left. **(B)** Corresponding heatmap to panel A, showing significance of the enrichment estimates (right-tailed, Binomial P-value, n=7,200 unique eVariants). **(C)** A lollipop plot showing the pronounced enrichment of lead eQTLs in the DHSs (DNAse Hypersensitivity Sites) across 53 cell/tissue types (colored as in A). We note a marked enrichment in DHS of blood cell types with lymphoblastoid cell line GM12878 as one of the top hits. **(D)** Volcano plot representing the enrichment analysis of lead eQTLs at TFBSs (Transcription factor binding sites) of 338 TF ChIP-seq binding profiles sourced from ENCODE. Data points reflecting a Bonferroni-corrected p-value < 0.001 and log2(fold-enrichment) > 1 stand out in red, underscoring those transcription factors where lead cis-eQTL enrichment is both statistically significant and of notable magnitude.

Functional annotation of one such eQTL signal that did not replicate in GTEx is shown in **Fig. S20**. The variant rs115070172 is associated with decreased expression of *GSTP1* and is largely private to the AMR continental group, as shown in **Fig. 5B**[59].

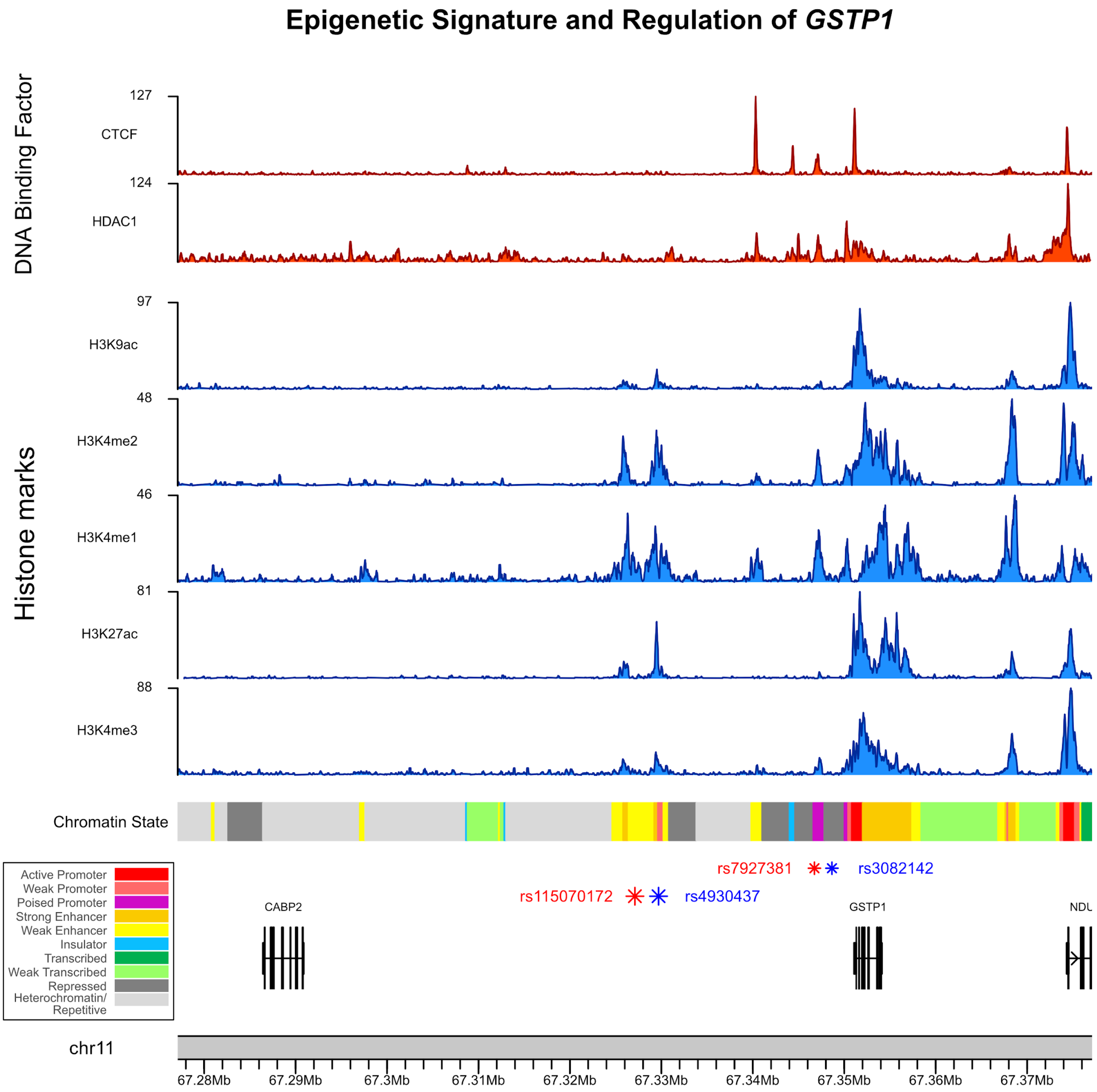

Figure S20. Functional epigenetic annotation of *GSTP1* eQTL credible signals. ENCODE epigenetic signals at the *GSTP1* locus, within lymphoblastoid cell line GM12878. From top down: 1) Red colored tracks denote binding of DNA associated factors. 2) Blue colored tracks show histone mark signals, including promoter and enhancer associated chromatin marks. 3) The multi-colored track shows the predicted chromatin state along the chromosome, legend at bottom left. 4) Asterisks represent fine-mapped eQTLs for *GSTP1*. The two red asterisks represent the lead eQTLs of the two *GSTP1* credible sets and the blue asterisks represent the corresponding SNP with the next highest PIP within the same credible set. The top two (smaller) asterisks represent eQTLs from one credible set, the bottom two (larger) asterisks represent eQTLs from the other credible set (which was highlighted in **Fig. 5**). 5) Gene annotation at and around the *GSTP1* locus.

17 Relationship between fixation index and differential gene expression

Weir & Cockerham's F_{ST}[44] was calculated for each autosomal fine-mapped lead eQTL identified in section **10.5** using the statistic's implementation in vcflib (version 1.0.0_rc2)[100]. For each lead eQTL, F_{ST} was estimated for each of the five target continental groups, where foreground samples fell within the target population (e.g., AFR) and background samples fell within any of the remaining four continental groups (e.g., EUR, SAS, EAS, or AMR). An average F_{ST} per eGene was calculated for each population by computing the mean of all eQTL F_{ST}'s identified for each respective eGene (negative F_{ST} values were converted to zeroes).

18 *cis*-eQTL effect size heterogeneity between populations

18.1 Modeling interaction effects between genotype and continental group

We discovered *cis*-eQTLs exhibiting effect size heterogeneity across continental groups (he-eQTLs) for each fine-mapped eGene. We first discovered he-eQTLs using a "single causal variant" model. For each eGene with at least one SuSiE credible set (described in section **10.5**), we selected a single variant with the lowest p-value in the FastQTL nominal pass (described in section **10.4**) to test for effect size heterogeneity. To ensure that we are detecting robust signals, we first filter to only those variants with MAF ≥ 0.05 in at least two continental groups. After filtering, 8,376/9,807 eGenes remained for analysis. From this set, we used the following approach to discover he-eQTLs.

For each eQTL we fit two models. We first fit a model regressing normalized TMM values onto sample genotype and eQTL mapping covariates (sex, top 5 genotyping PCs, 60 PEER factors). This is described in model **[1]** below, and we note that this is equivalent to the model used for nominal eQTL mapping with FastQTL (described in section **10.4**). Here, g_j describes the sample genotypes at the top nominal eQTL for gene *j*, E_j describes the inverse normal transformed TMM values of gene *j*, and X_{sex}, X_{PCA}, and X_{PEER} describe the covariates used for mapping. Next, we fit a model identical to model **[1]** but that now includes an additional genotype-by-continental group interaction term, as described in model **[2]**. Here, X_{CG} describes the continental groups of the samples. We performed an F-test to determine if the more complex model **[2]** explains the data significantly better than model **[1]**. We define he-eQTLs as those variants with significant F-statistics after Bonferroni correction ($p < 6 \times 10^{-6}$).

$$\textbf{(1)}\ \ E_j \sim g_j + X_{CG} + X_{sex} + X_{PCA} + X_{PEER}$$

$$\textbf{(2)}\ \ E_j \sim g_j + (g_j \times X_{CG}) + X_{CG} + X_{sex} + X_{PCA} + X_{PEER}$$

Given the stratification in the number of he-eQTLs discovered based on the number of causal signals SuSiE identified (**Fig. 5F**), we hypothesized that failing to control for multiple causal signals may lead to spurious discovery of he-eQTLs in the "single causal variant" model. To test this hypothesis, we employed a second approach to identify he-eQTLs. This approach mirrors the first with one important distinction: for each gene with multiple causal signals (i.e. multiple SuSiE credible sets), all lead fine-mapped eQTLs for that gene were included in the regression. This effectively controls for the additive effects of multiple causal SNPs. So, for each SuSiE lead eQTL, we first fit a model regressing normalized TMM values onto sample genotypes for the focal top hit variant and all other top hit variants for that gene (regardless of MAF), along with eQTL mapping covariates. This is described in model **[3]** below, assuming *n* credible sets for the focal gene. All variables are as described above, with $g_{j,i}$ being the genotypes of the focal variant (i.e. the genotype of the *i*th credible set for gene *j*). We next fit a model identical to model **[3]** but that now includes an additional genotype-by-continental group interaction term *for the focal variant only*. This is described in model **[4]**. As before, we performed an F-test to determine if model **[4]** explains the data significantly better than model **[3]**, and we define he-eQTLs as those variants with significant F-statistics after Bonferroni correction ($p < 4 \times 10^{-6}$).

$$\textbf{(3)}\ E_j \sim g_{j,1} + \ldots + g_{j,i} + \ldots + g_{j,n} + X_{CG} + X_{sex} + X_{PCA} + X_{PEER}$$

$$\textbf{(4)}\ E_j \sim g_{j,1} + \ldots + g_{j,i} + \ldots + g_{j,n} + (g_{j,i} \times X_{CG}) + X_{CG} + X_{sex} + X_{PCA} + X_{PEER}$$

Models 1-4 were fit using the `formula.api.ols` function from the *statsmodels* package (version 0.14.0) in Python. The F-test between models 1 and 2 and between models 3 and 4 was performed using the `stats.anova.anova_lm` function from the *statsmodels* package.

While continental group labels correlate with global ancestry proportions, these labels are proxies and we were curious whether more directly testing for interaction effects with global ancestry would yield distinct results. To address this question, we repeated the he-eQTL analysis, testing instead for genotype-by-global genotype PC interactions for each of the top five PCs (i.e. the same PCs included as covariates in the regression). No MAF thresholds were implemented for this analysis. The results of this analysis are presented in **Extended Data Fig. 7** and are qualitatively similar to the results of he-eQTL analysis using continental group labels presented in **Fig. 5F**.

18.2 Stratified *cis*-eQTL mapping and effect size estimation

One of the inherent assumptions of eQTL mapping and fine-mapping is that each causal variant has a single effect size that does not vary between subsets of the sample. This assumption may bias us towards discovering eQTLs *without* effect size differences between groups and may provide an alternative explanation for the lack of he-eQTLs discovered in our analysis of nominal and fine-mapped eQTLs in the previous section (section **18.1**).

To address this possibility, we performed *cis*-eQTL mapping, fine-mapping, and effect size estimation separately within each of the continental groups represented in MAGE. For the resulting credible sets, we then compared $\log_2$(aFC) effect sizes between continental groups to ask whether the effects of causal variants remain consistent, even when eQTL discovery and effect size estimation was performed independently within each continental group.

For each continental group, we followed the *cis*-eQTL mapping and fine-mapping pipeline described in section **10**. Within a continental group, TMM values from the full MAGE data set were extracted and inverse normal transformed and 30 PEER factors were calculated from those values, based on GTEx optimizations for sample sizes between 150-250 (MAGE continental group sample sizes range from 113-196). For consistency across continental groups, we did not recalculate the genotype PCs within each continental group, instead subsetting the PCs from the full MAGE data set (section **10.2**). We used these recalculated PEER factors, as well as genotype PCs and sex as covariates to perform eQTL mapping and fine-mapping for all 15,022 autosomal eGenes discovered from data set-wide nominal eQTL mapping.

We compared $\log_2$(aFC) effect sizes of fine-mapped credible sets between each pair of continental groups. For each such pair, we first identified shared causal signals between the two groups. We defined shared causal signals as a pair of credible sets where at least one variant was shared between the two credible sets and where each credible set *only* intersected the other credible set in the pair. Using this approach, we found that 25.4% to 52.9% of credible sets were comparable (i.e., uniquely shared variants) between continental groups (**Fig. S21**).

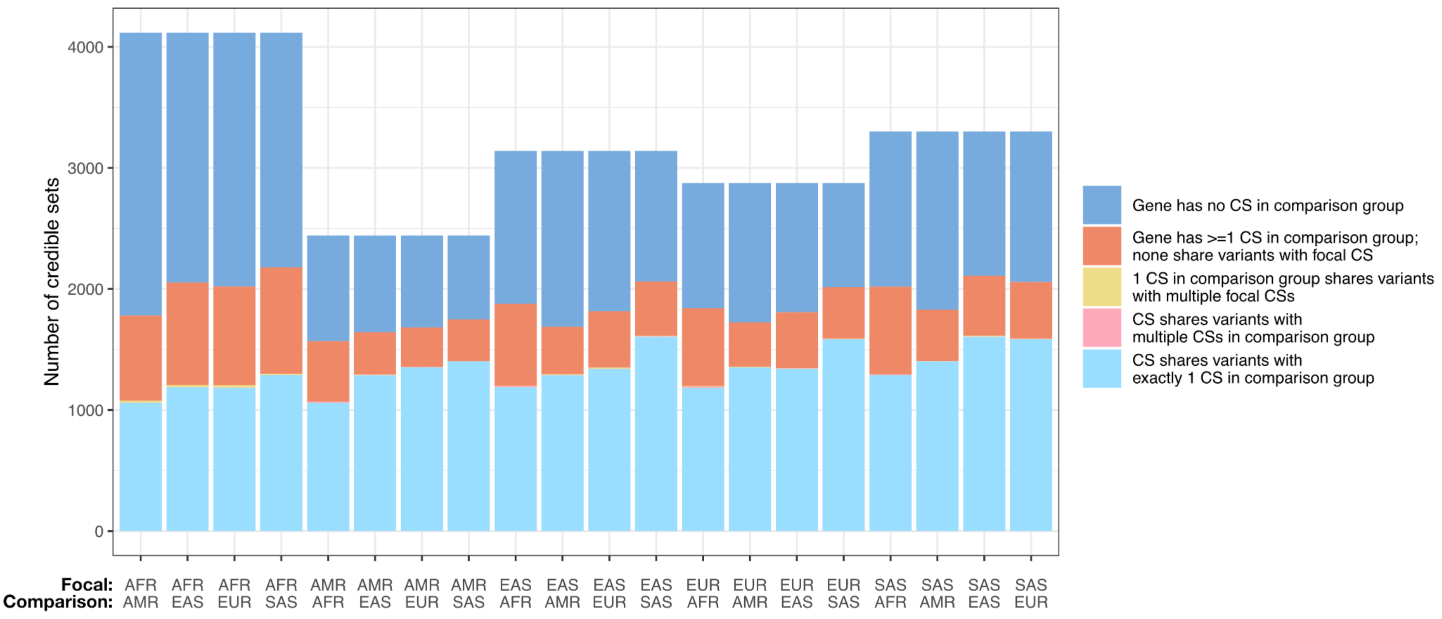

Figure S21. Number of shared causal signals between continental groups. Each bar represents all fine-mapped credible sets (CSs) identified within the focal continental group. CSs are colored by whether they share variants with a credible set in the comparison continental group. We compared log2(aFC) effect sizes only for CSs that shared variants with exactly one CS in the comparison group (light blue).

Because the lead eQTL of the two credible sets in a shared causal signal may not be the same, negative LD between the two lead eQTLs (i.e., the reference allele of the first lead eQTL is tightly linked with the alternative allele of the second lead eQTL) may lead to inverted effect size estimates and apparent effect size heterogeneity. This does not reflect a biological difference in the effect size of the underlying causal signal, but instead reflects the choice of effect allele on a causal haplotype. To address this, for a given credible set, we calculated LD between the lead eQTL and a variant that was shared between the credible sets of both continental groups. When the sign of LD was opposite in the two continental groups, we inverted the sign of the effect size in one of the continental groups.

For every pair of shared causal signals, we performed a two-sided Welch's t-test using the $\log_2$(aFC) (corrected for negative LD, described above) and standard error estimates from aFC-n to determine whether the effect size of that signal was significantly different in the two continental groups. To perform the Welch's t-test, we calculated the t-statistic t and degrees of freedom v as follows:

$$t = \frac{\bar{X}_1 - \bar{X}_2}{\sqrt{s_{\bar{X}_1}^2 + s_{\bar{X}_2}^2}}$$

$$v = \frac{\left(\frac{{s_1}^2}{N_1} + \frac{{s_2}^2}{N_2}\right)^2}{\frac{{s_1}^4}{{N_1}^2 v_1} + \frac{{s_2}^4}{{N_2}^2 v_2}},$$

where $\bar{X}_i$ describes the estimated $\log_2$(aFC) of the causal signal in the ith continental group, $s_{\bar{X}_i}$ is the standard error of that estimate from aFC-n, N_i is the continental group sample size, v_i is the degrees of freedom calculated as $v_i = N_i - 1$, and s_i is the sample standard deviation calculated as $s_i = s_{\bar{X}_i} * \sqrt{N_i}$. We used these values of t and v to calculate a p-value for the hypothesis that the effect size of the signal is different in the two continental groups.

We performed Bonferroni correction across all continental groups to account for multiple testing. We observed that between 97.5% (AFR vs. EUR) and 99.8% (AMR vs. SAS) of causal signals did not have significantly different effect sizes between continental groups (**Extended Data Fig. 8**). These results corroborate our original finding that effect size

heterogeneity is extremely rare among eQTLs and show that this pattern remains consistent even when explicitly allowing effect sizes to vary between continental groups during eQTL discovery.

References:

1. Li, Y. I. *et al.* RNA splicing is a primary link between genetic variation and disease. *Science* **352**, 600–604 (2016).
2. Brem, R. B., Yvert, G., Clinton, R. & Kruglyak, L. Genetic dissection of transcriptional regulation in budding yeast. *Science* **296**, 752–755 (2002).
3. Morley, M. *et al.* Genetic analysis of genome-wide variation in human gene expression. *Nature* **430**, 743–747 (2004).
4. Lappalainen, T. *et al.* Transcriptome and genome sequencing uncovers functional variation in humans. *Nature* **501**, 506–511 (2013).
5. GTEx Consortium. Genetic effects on gene expression across human tissues. *Nature* **550**, 204–213 (2017).
6. The 1000 Genomes Project Consortium *et al.* A global reference for human genetic variation. *Nature* **526**, 68–74 (2015).
7. Popejoy, A. B. & Fullerton, S. M. Genomics is failing on diversity. *Nature* **538**, 161–164 (2016).
8. Wojcik, G. L. *et al.* Genetic analyses of diverse populations improves discovery for complex traits. *Nature* **570**, 514–518 (2019).
9. Martin, A. R. *et al.* Human Demographic History Impacts Genetic Risk Prediction across Diverse Populations. *Am. J. Hum. Genet.* **107**, 788–789 (2020).
10. Kita, R., Venkataram, S., Zhou, Y. & Fraser, H. B. High-resolution mapping of cis-regulatory variation in budding yeast. *Proc Natl Acad Sci U S A* . **114**, E10736–E10744 (2017).
11. Storey, J. D. *et al.* Gene-expression variation within and among human populations. *Am. J. Hum. Genet.* **80**, 502–509 (2007).
12. Stranger, B. E. *et al.* Patterns of cis regulatory variation in diverse human populations. *PLoS Genet.* **8**, e1002639 (2012).
13. Martin, A. R. *et al.* Transcriptome sequencing from diverse human populations reveals differentiated regulatory architecture. *PLoS Genet.* **10**, e1004549 (2014).
14. Mogil, L. S. *et al.* Genetic architecture of gene expression traits across diverse populations. *PLoS Genet.* **14**, e1007586 (2018).
15. Kachuri, L. *et al.* Gene expression in African Americans, Puerto Ricans and Mexican Americans reveals ancestry-specific patterns of genetic architecture. *Nat. Genet.* **55**, 952–963 (2023).
16. Carlson, J., Henn, B. M., Al-Hindi, D. R. & Ramachandran, S. Counter the weaponization of genetics research by extremists. *Nature* **610**, 444–447 (2022).
17. DeGorter, M. K. *et al.* Transcriptomics and chromatin accessibility in multiple African population samples. *bioRxiv*.
18. Li, Y. I. *et al.* Annotation-free quantification of RNA splicing using LeafCutter. *Nat. Genet.* **50**, 151–158 (2018).
19. Lewontin, R. C. in *Evolutionary Biology* (eds Dobzhansky, T. et al.) 381–398 (Springer US, 1972).
20. Jorde, L. B. *et al.* The distribution of human genetic diversity: a comparison of mitochondrial, autosomal, and Y-chromosome data. *Am. J. Hum. Genet.* **66**, 979–988 (2000).
21. Bergström, A. *et al.* Insights into human genetic variation and population history from 929 diverse genomes. *Science* **367**, (2020).
22. Ramachandran, S. *et al.* Support from the relationship of genetic and geographic distance in human populations for a

serial founder effect originating in Africa. *Proc. Natl. Acad. Sci. U. S. A.* **102**, 15942–15947 (2005).

23. Prugnolle, F., Manica, A. & Balloux, F. Geography predicts neutral genetic diversity of human populations. *Curr. Biol.* **15**, R159–60 (2005).

24. Byrska-Bishop, M. *et al.* High-coverage whole-genome sequencing of the expanded 1000 Genomes Project cohort including 602 trios. *Cell* **185**, 3426–3440.e19 (2022).

25. Zou, Y., Carbonetto, P., Wang, G. & Stephens, M. Fine-mapping from summary data with the 'Sum of Single Effects' model. *PLoS Genet.* **18**, e1010299 (2022).

26. The GTEx Consortium. The GTEx Consortium atlas of genetic regulatory effects across human tissues. *Science* **369**, 1318–1330 (2020).

27. Jansen, R. *et al.* Conditional eQTL analysis reveals allelic heterogeneity of gene expression. *Hum. Mol. Genet.* **26**, 1444–1451 (2017).

28. Mohammadi, P., Castel, S. E., Brown, A. A. & Lappalainen, T. Quantifying the regulatory effect size of cis-acting genetic variation using allelic fold change. *Genome Res.* **27**, 1872–1884 (2017).

29. Huang, Q. Q., Ritchie, S. C., Brozynska, M. & Inouye, M. Power, false discovery rate and Winner's Curse in eQTL studies. *Nucleic Acids Res.* **46**, e133 (2018).

30. Lek, M. *et al.* Analysis of protein-coding genetic variation in 60,706 humans. *Nature* **536**, 285–291 (2016).

31. Glassberg, E. C., Gao, Z., Harpak, A., Lan, X. & Pritchard, J. K. Evidence for Weak Selective Constraint on Human Gene Expression. *Genetics* **211**, 757–772 (2019).

32. The Roadmap Epigenomics Consortium *et al.* Integrative analysis of 111 reference human epigenomes. *Nature* **518**, 317–330 (2015).

33. The ENCODE Project Consortium. An integrated encyclopedia of DNA elements in the human genome. *Nature* **489**, 57–74 (2012).

34. Wallace, C. A more accurate method for colocalisation analysis allowing for multiple causal variants. *PLoS Genet.* **17**, e1009440 (2021).

35. Giambartolomei, C. *et al.* Bayesian test for colocalisation between pairs of genetic association studies using summary statistics. *PLoS Genet.* **10**, e1004383 (2014).

36. Stapley, R. J. *et al.* Rare missense variants in Tropomyosin-4 (TPM4) are associated with platelet dysfunction, cytoskeletal defects, and excessive bleeding. *J. Thromb. Haemost.* **20**, (2022).

37. Hou, K. *et al.* Causal effects on complex traits are similar for common variants across segments of different continental ancestries within admixed individuals. *Nat. Genet.* **55**, 549–558 (2023).

38. Patel, R. A. *et al.* Genetic interactions drive heterogeneity in causal variant effect sizes for gene expression and complex traits. *Am. J. Hum. Genet.* **109**, 1286–1297 (2022).

39. Visscher, P. M. *et al.* 10 Years of GWAS Discovery: Biology, Function, and Translation. *Am. J. Hum. Genet.* **101**, 5–22 (2017).

40. Gutenkunst, R. N., Hernandez, R. D., Williamson, S. H. & Bustamante, C. D. Inferring the Joint Demographic History of Multiple Populations from Multidimensional SNP Frequency Data. *PLoS Genet.* **5**, (2009).

41. Fang, C. *et al.* Aberrant GSTP1 promoter methylation is associated with increased risk and advanced stage of breast cancer: a meta-analysis of 19 case-control studies. *BMC Cancer* **15**, 1–8 (2015).

42. Louie, S. M. *et al.* GSTP1 Is a Driver of Triple-Negative Breast Cancer Cell Metabolism and Pathogenicity. *Cell Chemical Biology* **23**, 567–578 (2016).

43. Arai, T. *et al.* Association of GSTP1 CpG Islands Hypermethylation with Poor Prognosis in Human Breast Cancers. *Breast Cancer Res. Treat.* **100**, 169–176 (2006).

44. Weir, B. S. & Cockerham, C. C. ESTIMATING F-STATISTICS FOR THE ANALYSIS OF POPULATION STRUCTURE. *Evolution* **38**, 1358–1370 (1984).

45. Saitou, M., Dahl, A., Wang, Q. & Liu, X. Allele frequency differences of causal variants have a major impact on low cross-ancestry portability of PRS. *medRxiv* 2022.10.21.22281371 (2022) doi:10.1101/2022.10.21.22281371.

46. Rau, C. D. *et al.* Modeling epistasis in mice and yeast using the proportion of two or more distinct genetic backgrounds: Evidence for 'polygenic epistasis'. *PLoS Genet.* **16**, e1009165 (2020).

47. Weissbrod, O. *et al.* Leveraging fine-mapping and multipopulation training data to improve cross-population polygenic risk scores. *Nat. Genet.* **54**, 450–458 (2022).

48. Mostafavi, H., Spence, J. P., Naqvi, S. & Pritchard, J. K. Systematic differences in discovery of genetic effects on gene expression and complex traits. *Nat. Genet.* **55**, 1866-1875 (2023).

49. Cheung, V. G. *et al.* Natural variation in human gene expression assessed in lymphoblastoid cells. *Nat. Genet.* **33**, 422–425 (2003).

50. Strober, B. J. *et al.* Dynamic genetic regulation of gene expression during cellular differentiation. *Science* **364**, 1287–1290 (2019).

51. Workman, R. E. *et al.* Nanopore native RNA sequencing of a human poly(A) transcriptome. *Nat. Methods* **16**, 1297–1305 (2019).

52. Glinos, D. A. *et al.* Transcriptome variation in human tissues revealed by long-read sequencing. *Nature* **608**, 353–359 (2022).

53. Reese, F. *et al.* The ENCODE4 long-read RNA-seq collection reveals distinct classes of transcript structure diversity. *bioRxiv* (2023) doi:10.1101/2023.05.15.540865.

54. Claw, K. G. *et al.* A framework for enhancing ethical genomic research with Indigenous communities. *Nat. Commun.* **9**, 2957 (2018).

55. Liao, W.-W. *et al.* A draft human pangenome reference. *Nature* **617**, 312–324 (2023).

56. Sibbesen, J. A. *et al.* Haplotype-aware pantranscriptome analyses using spliced pangenome graphs. *Nat. Methods* **20**, 239–247 (2023).

57. The International HapMap Consortium. The International HapMap Project. *Nature* **426**, 789–796 (2003).

58. Alexander, D. H., Novembre, J. & Lange, K. Fast model-based estimation of ancestry in unrelated individuals. *Genome Res.* **19**, 1655–1664 (2009).

59. Marcus, J. H. & Novembre, J. Visualizing the geography of genetic variants. *Bioinformatics* **33**, 594–595 (2017).

60. Taylor. D & McCoy, R. MAGE: Multi-ancestry Analysis of Gene Expression v1.0. Zenodo https://doi.org/10.5281/zenodo.10535719 (2024).

61. Taylor, D., McCoy, R., Biddanda, A. & Tassia, M. mccoy-lab/MAGE: MAGE v.1.0.0. Zenodo https://doi.org/10.5281/zenodo.10072080 (2023).

62. Purcell, S. *et al.* PLINK: a tool set for whole-genome association and population-based linkage analyses. *Am. J. Hum. Genet.* **81**, 559–575 (2007).

63. Frankish, A. *et al.* GENCODE reference annotation for the human and mouse genomes. *Nucleic Acids Res.* **47**, D766–D773 (2019).

64. Patro, R., Duggal, G., Love, M. I., Irizarry, R. A. & Kingsford, C. Salmon provides fast and bias-aware quantification of transcript expression. *Nat. Methods* **14**, 417–419 (2017).

65. Soneson, C., Love, M. I. & Robinson, M. D. Differential analyses for RNA-seq: transcript-level estimates improve gene-level inferences. *F1000Res.* **4**, 1521 (2015).

66. Dobin, A. *et al.* STAR: ultrafast universal RNA-seq aligner. *Bioinformatics* **29**, 15–21 (2013).

67. Fairley, S., Lowy-Gallego, E., Perry, E. & Flicek, P. The International Genome Sample Resource (IGSR) collection of open human genomic variation resources. *Nucleic Acids Res.* **48**, D941–D947 (2019).

68. Degner, J. F. *et al.* Effect of read-mapping biases on detecting allele-specific expression from RNA-sequencing data. *Bioinformatics* **25**, 3207–3212 (2009).

69. Chen, N.-C., Solomon, B., Mun, T., Iyer, S. & Langmead, B. Reference flow: reducing reference bias using multiple population genomes. *Genome Biol.* **22**, 8 (2021).

70. Garrido-Martín, D., Calvo, M., Reverter, F. & Guigó, R. A fast non-parametric test of association for multiple traits. *Genome Biol.* **24**, 230 (2023).

71. Love, M. I., Huber, W. & Anders, S. Moderated estimation of fold change and dispersion for RNA-seq data with DESeq2. *Genome Biol.* **15**, 550 (2014).

72. Robinson, M. D. & Oshlack, A. A scaling normalization method for differential expression analysis of RNA-seq data. *Genome Biol.* **11**, R25 (2010).

73. Robinson, M. D., McCarthy, D. J. & Smyth, G. K. edgeR: a Bioconductor package for differential expression analysis of digital gene expression data. *Bioinformatics* **26**, 139–140 (2010).

74. Stegle, O., Parts, L., Piipari, M., Winn, J. & Durbin, R. Using probabilistic estimation of expression residuals (PEER) to obtain increased power and interpretability of gene expression analyses. *Nat. Protoc.* **7**, 500–507 (2012).

75. The International HapMap Consortium. A second generation human haplotype map of over 3.1 million SNPs. *Nature* **449**, 851–861 (2008).

76. The International HapMap 3 Consortium. Integrating common and rare genetic variation in diverse human populations. *Nature* **467**, 52–58 (2010).

77. Ongen, H., Buil, A., Brown, A. A., Dermitzakis, E. T. & Delaneau, O. Fast and efficient QTL mapper for thousands of molecular phenotypes. *Bioinformatics* **32**, 1479–1485 (2016).

78. Tukiainen, T. *et al.* Landscape of X chromosome inactivation across human tissues. *Nature* **550**, 244–248 (2017).

79. Wang, G., Sarkar, A., Carbonetto, P. & Stephens, M. A Simple New Approach to Variable Selection in Regression, with Application to Genetic Fine Mapping. *J. R. Stat. Soc. Series B Stat. Methodol.* **82**, 1273–1300 (2020).

80. Ehsan, N. *et al.* Haplotype-aware modeling of cis-regulatory effects highlights the gaps remaining in eQTL data. *Nat. Commun.* **15**, 522 (2024).

81. Petrovski, S., Wang, Q., Heinzen, E. L., Allen, A. S. & Goldstein, D. B. Genic intolerance to functional variation and the interpretation of personal genomes. *PLoS Genet.* **9**, e1003709 (2013).

82. Karczewski, K. J. *et al.* The mutational constraint spectrum quantified from variation in 141,456 humans. *Nature* **581**, 434–443 (2020).

83. Collins, R. L. *et al.* A cross-disorder dosage sensitivity map of the human genome. *Cell* **185**, 3041–3055.e25 (2022).

84. Agarwal, I., Fuller, Z. L., Myers, S. R. & Przeworski, M. Relating pathogenic loss-of-function mutations in humans to their evolutionary fitness costs. *Elife* **12**, (2023).

85. Chen, S. *et al.* A genomic mutational constraint map using variation in 76,156 human genomes. *Nature* **625**, 92–100 (2024).

86. Siepel, A. *et al.* Evolutionarily conserved elements in vertebrate, insect, worm, and yeast genomes. *Genome Res.* **15**, 1034–1050 (2005).

87. Armstrong, J. *et al.* Progressive Cactus is a multiple-genome aligner for the thousand-genome era. *Nature* **587**, 246–251 (2020).

88. Kuderna, L. F. K. *et al.* A global catalog of whole-genome diversity from 233 primate species. *Science* **380**, 906–913 (2023).

89. Morales, J. *et al.* A joint NCBI and EMBL-EBI transcript set for clinical genomics and research. *Nature* **604**, 310–315 (2022).

90. The ENCODE Project Consortium *et al.* Expanded encyclopaedias of DNA elements in the human and mouse genomes. *Nature* **583**, 699–710 (2020).

91. Lawrence, M. *et al.* Software for computing and annotating genomic ranges. *PLoS Comput. Biol.* **9**, e1003118 (2013).

92. Quinlan, A. R. & Hall, I. M. BEDTools: a flexible suite of utilities for comparing genomic features. *Bioinformatics* **26**, 841–842 (2010).

93. Schmidt, E. M. *et al.* GREGOR: evaluating global enrichment of trait-associated variants in epigenomic features using a systematic, data-driven approach. *Bioinformatics* **31**, 2601–2606 (2015).

94. Ernst, J. & Kellis, M. ChromHMM: automating chromatin-state discovery and characterization. *Nat. Methods* **9**, 215–216 (2012).

95. McLaren, W. *et al.* The Ensembl Variant Effect Predictor. *Genome Biol.* **17**, 122 (2016).

96. Sollis, E. *et al.* The NHGRI-EBI GWAS Catalog: knowledgebase and deposition resource. *Nucleic Acids Res.* **51**, D977–D985 (2023).

97. Taliun, D. *et al.* Sequencing of 53,831 diverse genomes from the NHLBI TOPMed Program. *Nature* **590**, 290–299 (2021).

98. Biddanda, A., Rice, D. P. & Novembre, J. A variant-centric perspective on geographic patterns of human allele frequency variation. *Elife* **9**, (2020).

99. Wen, X., Lee, Y., Luca, F. & Pique-Regi, R. Efficient Integrative Multi-SNP Association Analysis via Deterministic Approximation of Posteriors. *Am. J. Hum. Genet.* **98**, 1114–1129 (2016).

100. Garrison, E., Kronenberg, Z. N., Dawson, E. T., Pedersen, B. S. & Prins, P. A spectrum of free software tools for processing the VCF variant call format: vcflib, bio-vcf, cyvcf2, hts-nim and slivar. *PLoS Comput. Biol.* **18**, e1009123 (2022).
