# Supplementary material for: Sources of gene expression variation in a globally diverse human cohort
Source: Nature. 2024 Jul 17;632(8023):122–30. doi: 10.1038/s41586-024-07708-2 (PMC11291278; doi:10.1038/s41586-024-07708-2)
Supplement: Supplementary file 2 — Reporting Summary [file 41586_2024_7708_MOESM2_ESM.pdf]

nature portfolio

Corresponding author(s): McCoy, R. C.

Last updated by author(s): May 20, 2024

Reporting Summary

Nature Portfolio wishes to improve the reproducibility of the work that we publish. This form provides structure for consistency and transparency in reporting. For further information on Nature Portfolio policies, see our Editorial Policies and the Editorial Policy Checklist.

Statistics

For all statistical analyses, confirm that the following items are present in the figure legend, table legend, main text, or Methods section.

n/a	Confirmed	
☐	☒	The exact sample size (*n*) for each experimental group/condition, given as a discrete number and unit of measurement
☐	☒	A statement on whether measurements were taken from distinct samples or whether the same sample was measured repeatedly
☐	☒	The statistical test(s) used AND whether they are one- or two-sided<br>*Only common tests should be described solely by name; describe more complex techniques in the Methods section.*
☐	☒	A description of all covariates tested
☐	☒	A description of any assumptions or corrections, such as tests of normality and adjustment for multiple comparisons
☐	☒	A full description of the statistical parameters including central tendency (e.g. means) or other basic estimates (e.g. regression coefficient) AND variation (e.g. standard deviation) or associated estimates of uncertainty (e.g. confidence intervals)
☐	☒	For null hypothesis testing, the test statistic (e.g. *F*, *t*, *r*) with confidence intervals, effect sizes, degrees of freedom and *P* value noted<br>*Give P values as exact values whenever suitable.*
☒	☐	For Bayesian analysis, information on the choice of priors and Markov chain Monte Carlo settings
☒	☐	For hierarchical and complex designs, identification of the appropriate level for tests and full reporting of outcomes
☐	☒	Estimates of effect sizes (e.g. Cohen's *d*, Pearson's *r*), indicating how they were calculated

Our web collection on statistics for biologists contains articles on many of the points above.

Software and code

Policy information about availability of computer code

Data collection

No software was used for data collection.

Data analysis

All scripts and commands used to prepare and analyze the data can be found in a public GitHub repository (https://github.com/mccoy-lab/MAGE) and stable Zenodo repository (doi: 10.5281/zenodo.10072080) and are described in the Methods section.

The data analysis packages/tools used in this manuscript are as follows:
* ADMIXTURE tool (version 1.3.0)
* Plink tool (version 1.90b6.21)
* Salmon tool (version 1.5.2)
* tximport R package (version 1.18.0 and 1.24.0)
* STAR tool (version 2.7.10a)
* Leafcutter tool (version 0.2.9)
* regtools tool (version 0.5.2)
* MANTA R package (version 1.0.0)
* car R package (verzion 3.1-2)
* DESeq2 R package (version 1.36.0)
* stats R package (version 4.3.0)
* lme4 R package (version 1.1-34)
* EdgeR R package (version 3.32.1)
* peertool tool (version 1.0)
* FastQTL tool (version 2.184_gtex)

* susieR R package (version 0.12.16)
* aFC-n tool (version 1.0.0 modified in-house, available here: https://github.com/dtaylo95/aFCn)
* GenomicRanges R package (version 1.38.0)
* bedtools tool (version 2.29.2)
* GREGOR tool (version 1.3.1)
* Ensembl Variant Effect Predictor (VEP) tool (version 109)
* LOFTEE plug-in for VEP (version 1.0.2)
* coloc R package (version 5.2.3)
* geovar python package (version 1.0.2)
* vcflib tool (version 1.0.0_rc2)
* statsmodels python package (version 0.14.0)

For manuscripts utilizing custom algorithms or software that are central to the research but not yet described in published literature, software must be made available to editors and reviewers. We strongly encourage code deposition in a community repository (e.g. GitHub). See the Nature Portfolio guidelines for submitting code & software for further information.

Data

Policy information about availability of data

All manuscripts must include a data availability statement. This statement should provide the following information, where applicable:

- Accession codes, unique identifiers, or web links for publicly available datasets
- A description of any restrictions on data availability
- For clinical datasets or third party data, please ensure that the statement adheres to our policy

All sequencing data is available from the NCBI Sequence Read Archive (Accession: PRJNA851328). Processed gene expression matrices and QTL mapping results are available on Zenodo (doi: 10.5281/zenodo.10535719).

External datasets used:
* GRCh38 reference genome (https://www.ncbi.nlm.nih.gov/datasets/genome/GCA_000001405.15/)
* Sample genotypes from the New York Genome Center (NYGC) high-coverage WGS of the 1000 Genomes Project (1KGP; https://ftp.1000genomes.ebi.ac.uk/vol1/ftp/data_collections/1000G_2504_high_coverage/working/20201028_3202_phased/; https://www.internationalgenome.org/data-portal/data-collection/30x-grch38)
* Sex, continental group, and population labels for 1KGP samples (filtered to 30x GRCh38 samples; https://www.internationalgenome.org/data-portal/sample)
* Sample genotypes for GTEx v9 (dbGaP accession phs000424.v9.p2; https://www.ncbi.nlm.nih.gov/projects/gap/cgi-bin/study.cgi?study_id=phs000424.v9.p2)
* Transcript sequences and gene annotations from GENCODE v38 (https://www.gencodegenes.org/human/release_38.html)
* pLI and LOEUF (https://gnomad.broadinstitute.org/downloads#v4-constraint)
* pHaplo and pTriplo (https://zenodo.org/records/6347673)
* hs (https://github.com/agarwal-i/loss-of-function-fitness-effects)
* RVIS (https://doi.org/10.1371/journal.pgen.1003709.s002)
* PhyloP (https://hgdownload.soe.ucsc.edu/goldenPath/hg38/phyloP447way/)
* ENCODE TF binding sites (https://genome.ucsc.edu/cgi-bin/hgTrackUi?db=hg38&g=encRegTfbsClustered)
* Roadmap Epigenomics chromHMM and DHS annotations (https://egg2.wustl.edu/roadmap/web_portal/chr_state_learning.html)
* GWAS Catalog harmonized GWAS summary statistics from the PAGE study (https://www.ebi.ac.uk/gwas/publications/31217584)
* Variant calls for samples in the PAGE study are part of TOPMED (dbGaP accession: phs001974.v5.p1; https://www.ncbi.nlm.nih.gov/projects/gap/cgi-bin/study.cgi?study_id=phs001974.v5.p1)
* GTEx v8 eQTL DAPG fine-mapping results (https://www.gtexportal.org/home/downloads/adult-gtex/qtl)

Research involving human participants, their data, or biological material

Policy information about studies with human participants or human data. See also policy information about sex, gender (identity/presentation), and sexual orientation and race, ethnicity and racism.

Reporting on sex and gender	Sex (as reported previously by the 1000 Genomes Project) was used as a covariate in all QTL mapping analyses. However, all samples are considered together, regardless of sex, for all analyses (i.e., analyses are not sex-stratified, but are sex-adjusted).
Reporting on race, ethnicity, or other socially relevant groupings	Samples were previously assigned to 1) populations and 2) continental groups (which comprise multiple populations) by the 1000 Genomes Project based on sampling location and expected patterns of genetic ancestry. We used these same continental group and population labels in this study.<br><br>For most analyses, samples were analyzed together, regardless of population label. For QTL mapping, the top 5 genotype PCs (which are found to correlate with population and continental group labels) were used as covariates to control for trans effects driven by global ancestry proportions.
Population characteristics	Initial sample collection was performed previously by the 1000 Genomes Project. All individuals were reported to be healthy adults at the time of sample collection. Blood was collected from each sample and was EBV-transformed to establish lymphoblastoid cell lines. 30x whole genome sequencing for each of these cell lines is available from the New York Genome Center, and we generated RNA-seq data from these cell lines in this study.
Recruitment	N/A - Recruitment performed as part of earlier study by the 1000 Genomes Consortium.
Ethics oversight	The Johns Hopkins Homewood IRB deemed this work not to meet the definition of human subjects research (HIRB00009187).

Note that full information on the approval of the study protocol must also be provided in the manuscript.

Field-specific reporting

Please select the one below that is the best fit for your research. If you are not sure, read the appropriate sections before making your selection.

☒ Life sciences ☐ Behavioural & social sciences ☐ Ecological, evolutionary & environmental sciences

For a reference copy of the document with all sections, see nature.com/documents/nr-reporting-summary-flat.pdf

Life sciences study design

All studies must disclose on these points even when the disclosure is negative.

Sample size	Sample size was chosen based on previous results by the GTEx consortium that demonstrated high power to detect eQTL associations at low MAF with 750 samples. We chose to sequence 780 libraries from 732 cell lines, such that we could sequence 24 cell lines in triplicate. The 732 cell lines were selected as evenly as possible from the 26 populations in the 1000 Genomes project. One library failed sequencing, leaving us with 779 total libraries across 731 unique cell lines.
Data exclusions	No data were excluded.
Replication	As described above, 24 cell lines were sequenced in triplicate. Library preparation and sequencing was successful for all replicates. Each of the replicated cell lines were sequenced twice in one sequencing batch, and a third time in a separate sequencing batch, to quantify within vs. between batch variation. We found that across the replicate libraries, the cell line that the library was generated from explained more variance in both expression level and splicing than the library batch (see Supplementary Information).
Randomization	Sequencing libraries were randomized across sequencing batches in a stratified manner: libraries were stratified across batches based on the population label of the cell line, to reduce confounding between sequencing batch and population.
Blinding	Not applicable: blinding was not relevant to experimental design or analysis. This is not a clinical study; there are no treatment or control groups. We are analyzing RNA-sequencing data from lymphoblastoid cell lines.

Reporting for specific materials, systems and methods

We require information from authors about some types of materials, experimental systems and methods used in many studies. Here, indicate whether each material, system or method listed is relevant to your study. If you are not sure if a list item applies to your research, read the appropriate section before selecting a response.

Materials & experimental systems

n/a	Involved in the study	
☒	☐	Antibodies
☐	☒	Eukaryotic cell lines
☒	☐	Palaeontology and archaeology
☒	☐	Animals and other organisms
☒	☐	Clinical data
☒	☐	Dual use research of concern
☒	☐	Plants

Methods

n/a	Involved in the study	
☒	☐	ChIP-seq
☒	☐	Flow cytometry
☒	☐	MRI-based neuroimaging

Eukaryotic cell lines

Policy information about cell lines and Sex and Gender in Research

Cell line source(s)	All cell lines are lymphoblastoid cell lines (LCLs) procured from the Coriell Insitute for Medical Research. Cell line sex is previously reported by the 1000 Genomes Project.
Authentication	Quality control procedures were performed according to standard practices of the NHGRI Repository at Coriell. Cell line identity was confirmed using a multiplex PCR assay for six autosomal microsatellite markers.
Mycoplasma contamination	At Coriell, cultures are tested and found free of mycoplasma, bacteria, and fungi during expansion, at the time of frozen storage, and after recovery of stock for distribution from liquid nitrogen.
Commonly misidentified lines (See ICLAC register)	Not applicable

Plants

Seed stocks	Not applicable
Novel plant genotypes	Not applicable
Authentication	Not applicable
